# Effective data visualization strategies in untargeted metabolomics†

**DOI:** 10.1039/d4np00039k

**Published:** 2024-12-02

**Authors:** Kevin Mildau, Henry Ehlers, Mara Meisenburg, Elena Del Pup, Robert A. Koetsier, Laura Rosina Torres Ortega, Niek F. de Jonge, Kumar Saurabh Singh, Dora Ferreira, Kgalaletso Othibeng, Fidele Tugizimana, Florian Huber, Justin J. J. van der Hooft

**Affiliations:** a Bioinformatics Group, Wageningen University & Research Wageningen The Netherlands kevin.mildau@wur.nl justin.vanderhooft@wur.nl; b Visualization Group, Institute of Visual Computing and Human-Centered Technology, TU Wien Vienna Austria henry.ehlers@tuwien.ac.at; c Adaptation Physiology Group, Wageningen University & Research Wageningen The Netherlands; d Maastricht University Faculty of Science and Engineering, Plant Functional Genomics Maastricht Limburg The Netherlands; e Faculty of Environment, Science and Economy, University of Exeter Penryl Cornwall UK; f NAICONS Srl Milan Italy; g Department of Biochemistry, University of Johannesburg Johannesburg South Africa; h Centre for Digitalisation and Digitality, Düsseldorf University of Applied Sciences Düsseldorf Germany

## Abstract

Covering: 2014 to 2023 for metabolomics, 2002 to 2023 for information visualization

LC-MS/MS-based untargeted metabolomics is a rapidly developing research field spawning increasing numbers of computational metabolomics tools assisting researchers with their complex data processing, analysis, and interpretation tasks. In this article, we review the entire untargeted metabolomics workflow from the perspective of information visualization, visual analytics and visual data integration. Data visualization is a crucial step at every stage of the metabolomics workflow, where it provides core components of data inspection, evaluation, and sharing capabilities. However, due to the large number of available data analysis tools and corresponding visualization components, it is hard for both users and developers to get an overview of what is already available and which tools are suitable for their analysis. In addition, there is little cross-pollination between the fields of data visualization and metabolomics, leaving visual tools to be designed in a secondary and mostly *ad hoc* fashion. With this review, we aim to bridge the gap between the fields of untargeted metabolomics and data visualization. First, we introduce data visualization to the untargeted metabolomics field as a topic worthy of its own dedicated research, and provide a primer on cutting-edge visualization research into data visualization for both researchers as well as developers active in metabolomics. We extend this primer with a discussion of best practices for data visualization as they have emerged from data visualization studies. Second, we provide a practical roadmap to the visual tool landscape and its use within the untargeted metabolomics field. Here, for several computational analysis stages within the untargeted metabolomics workflow, we provide an overview of commonly used visual strategies with practical examples. In this context, we will also outline promising areas for further research and development. We end the review with a set of recommendations for developers and users on how to make the best use of visualizations for more effective and transparent communication of results.

## Abstract 2

Untargeted metabolomics faces researchers with complex processing, analysis, and interpretation tasks. Here, we review computational tools from the perspective of information visualization and underpin its critical role and importance to the field.

## Graphical abstract

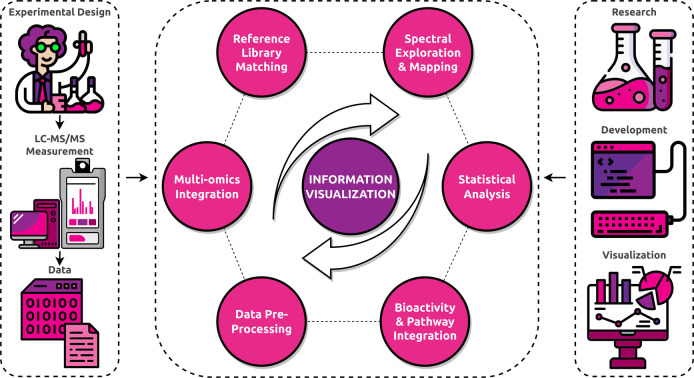

## 1. Background and motivation

The field of untargeted metabolomics is rapidly developing with new experimental and computational workflows appearing almost continuously. This is for a good reason; the datasets generated by LC-MS/MS metabolomics experiments are sizeable and abstract, requiring a myriad of processing steps and interconnected analysis steps to gain insights into the (bio)chemistry of the samples studied. Many of these processing steps come with numerous settings or tuning parameters which, while difficult to set in some optimal fashion, can have tremendous impacts on the resulting intermediate data produced. Each of the many workflow steps, such as the assessment of potential matrix effects and experimental data quality affirmation, the separation of signal from noise, the complex extraction and separation of features, the challenging cross sample alignment of features affected by retention time and mass shifts, the adjustment of intensities to take into account potential batch effects, the validity assessment of library matches or annotations, the complex MS/MS spectral data organization processes, all the way to cross-omics integration methods, come with impactful choices. It thus comes as no surprise that metabolomics researchers need to manually validate their pre-processing steps and conclusions at each step of their analysis. Untargeted metabolomics data analysis is hence a prime example of a research pipeline heavily dependent on expert “human in the loop” input. However, many of the data components such as multi-dimensional chromatographic outputs or MS/MS spectral data are notoriously difficult to interpret in their raw form. Hence, visualizations are being developed and integrated to facilitate many of these laborious and abstract tasks.

**Fig. 1:**
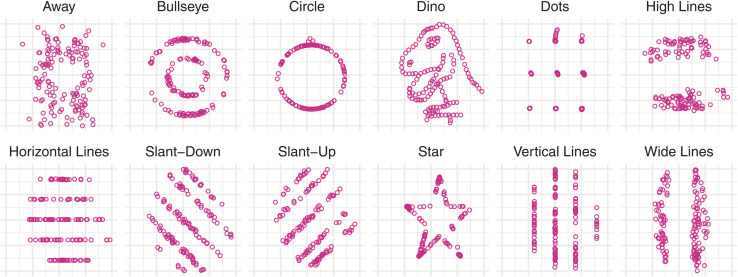
Scatter plots of twelve of the thirteen datasaurus datasets (see ESI† for X-shape variant).^1^ The datasaurus dataset is a constructed dataset intended to illustrate how misleading summary statistics and models outcomes can be, and how powerful visualization can be at showing the actual differences behind apparently similar overview statistics. Each dataset is constructed in such a way that summary statistics match those of a Dinosaur scatter plot drawing. Specifically, each dataset has near identical *X* and *Y* variable means and standard deviations, near identical correlation between *X* and *Y* variables, and, a linear model fitted to model *Y* as a function of *X* in each case has near identical intercepts and slope, as well as identical R squared summary statistics. This makes the datasets indistinguishable from the perspective of a comprehensive set of summary statistics. Visual inspection of the tables is also insufficient to grasp differences in the data. In the figure above, *X* and *Y* axes for all plots are identical and range from 0 to 100. Axis labels and tick marks are not shown as they are not essential.

Data visualization and the scientific process are heavily intertwined. As scientific data becomes increasingly complex and sizeable, statistical and visual strategies are increasingly combined to generate data overviews, navigate complex datasets, and gain specific insights. In addition, visualization is used in any field of science to report data and model ideas *via* infographics. As such, visualizations supplement, extend, and build upon statistical measures, but also allow assessing applicability or distortions caused by the latter (Fig. 1). Scientific visualizations also render insights more tangible, with a shared understanding of visualizations allowing scientists to rapidly build consensus understanding on the main insights from data, *e.g.*, volcano plots giving a snapshot view of treatment impacts and affected metabolites (*i.e.*, see Fig. 18 in Section 3.3).

The scientific community takes great care of validating and reporting analysis choices and final data uncertainties, often through a combination of statistical tabular data and data visualizations thereof. Inherently, science is a human-in-the-loop decision-making process. It is important to stress that reliable and predictable analysis steps can and should be dealt with using automated systems, allowing researchers to focus on parts that cannot be automated and require expert evaluation. For the latter, data analysis workflows but also scientist-to-scientist communication is facilitated and accelerated by visual tools. Visualizations are used as a means to augment researchers decision-making capabilities by summarizing data (*e.g.*, using boxplots or scatters with line graphs), extracting and highlighting patterns within the data (*e.g.*, through cluster heatmaps), and organizing and showcasing relations between data (*e.g.*, by network visualizations). Visualizations do so by extending the human users' cognitive abilities for how much information they can hold in memory by translating data to a more accessible and visual channel.^1^

While the sharing of informative infographics in scientific papers is an integral part of the scientific process, most scientific visualizations are rendered and consumed on computer displays. Indeed, modern visual strategies usually involve interactivity, allowing researchers to interact with and explore their data from different angles without having to manually re-generate different plots. This in-context exploration and analysis can streamline scientific discovery. In the age of big data, scientists are less confronted with ever larger tabular data, but rather with ever more sophisticated statistical and visual summaries. Even small tabular datasets are difficult to visually process, and statistical summaries may not be detailed enough to glance at true data patterns as seen in the example of the datasaurus and Anscombe datasets.^1^ Here, both tabular data and statistical summaries are insufficient to gain differentiating insights into the data which are immediately apparent when looking at the simple scatter line graphs (Fig. 1, and ESI†).^2–4^

**Fig. 2:**
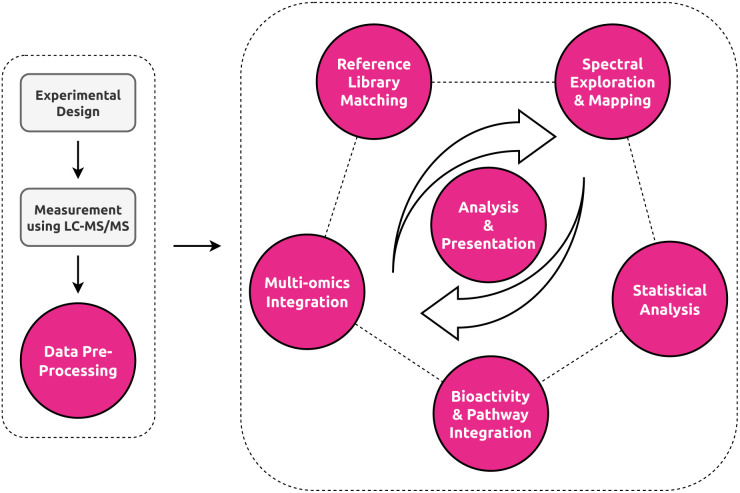
Overview of the untargeted metabolomics workflow. Color-highlighted nodes represent the data analysis stages and their visual components covered by this review. Analyses in untargeted metabolomics rarely follow a strict linear path, with different stages of the analyses happening in different order or not at all depending on the study. Central to all stages are stage-specific or inter-stage analysis and presentation visualizations.

**Fig. 3:**
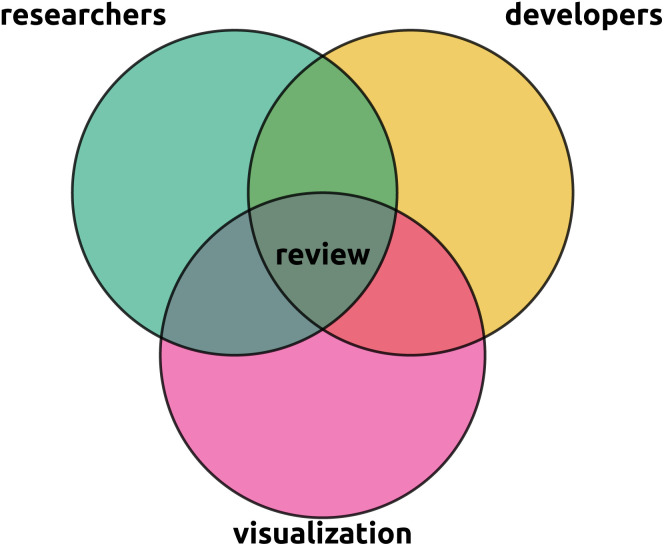
This review is situated in the intersection between the metabolomics researcher perspective, the metabolomics developer perspective, and the visualization field experts perspective. In particular, we aim to provide researchers with an overview of the visual capabilities available to them, provide developers with an overview of what possibilities and uncovered needs are, and visualization experts with an entry point to the current state of visualization in metabolomics. A primary aim of this review is to raise awareness of potential cross pollination of the visualization field into untargeted metabolomics. Rather than being comprehensive, we intend to provide an overview and highlight hot topics and refer to more focused reviews for details.

In this paper, we will cover three distinct sections to tackle the multifaceted and interdisciplinary nature of data visualization in untargeted metabolomics (Fig. 3). We here below describe the main aims and contents of each section to guide the reader in finding the most relevant sections based on their background and interest. First, we provide a primer and bird's eye roadmap to the field of information visualization and what the metabolomics field can learn from both well-established and cutting-edge research in this field. Here, we will deal with both general visualization and more specifically network visualization in light of its importance in the field for both exploratory data analysis and multi-omics integration. Second, we share a practical roadmap where we highlight for each stage of the untargeted metabolomics workflow (Fig. 2) several promising tools with respect to their visualization strategies and discuss potential untapped niches. We hope that this overview will help practitioners to find the right tool for the job, as well as developers by providing them with an idea of what is still lacking in the field. Finally, we establish a developer roadmap to provide the analysis and visual tool creators with practical recommendations to follow that we believe would lead to improved visualization workflows that enhance scientific insights.

## 2. Information visualization – a road map

Information visualization, sometimes shorted to InfoVis, is the study of how to best understand, explore, and analyze data to generate knowledge through interactive and exploratory visualizations.^5^ More specifically, the field concerns itself with five key areas (highlighted in bold below) that range from abstract model development through the empirical evaluation of visualization approaches to the engineering of novel (domain-specific) visualization solutions. First, researchers aim to **develop novel conceptual frameworks and models to describe and better understand the roles of data, users, analysis, and visualization in the interactive and non-linear process of knowledge generation**.^6^ These knowledge-generation endeavors are driven by particular domain-specific goals. Moving beyond domain goals, research aims to **describe, categorize, and understand the various types of goals and tasks** that underlie information visualization, on both a coarse^7^ and fine level.^8^ Using both conceptual knowledge generation models and analysis goal taxonomies, researchers **develop novel visualization approaches to facilitate target tasks and analysis goals**.^9^ As with any tool development, the developed visual encoding and visualization approaches need to be proved useful. Hence, an important pillar of information visualization is the **empirical evaluation and comparison of visualization approaches***via* task-driven quantitative user performance studies and user experience studies, sometimes augmented with additional qualitative analyses.^10^ Finally, building upon all aspects of information visualization, researchers work in tandem with domain experts to **develop novel, domain-specific visualization solutions** tackling the particular needs of experts in that field.^11^ In summary, putting all five pillars together, information visualization aims to develop, study, and evaluate different ways in which users can generate insight from as well as consume interactive data visualizations.^12^

Given its increasing importance in the visualization of ever larger datasets, information visualization has found use across a wide range of domains, including diverse fields such as the social sciences,^13^ text visualization,^14^ financial analysis,^15^ and of course, metabolomics.^16^ Unfortunately, the broad applicability and many rapidly evolving subdomains of information visualization make it daunting to approach for newcomers. Several books,^2,17^ surveys,^5,18,19^ and surveys of surveys^20^ have been published to allow the visualization community to better track the state-of-the-art. However, as these tend to be written by and for members of the visualization community, their findings and summaries rarely find practical application. Aiming to tackle this, there have been several calls for bringing together both visualization and domain experts to facilitate dissemination of theoretical insights of the information visualization field into practice. Examples hereof include the social sciences and visualization experts,^21^ statistics and visualization,^22^ and discussions of the importance of visualization for both analysis and communication in biology.^23^ Joining the effort, we aim to provide a newcomer-friendly introduction to information visualization to metabolomics experts and tool developers. We provide an overview of the field as well as key references as a roadmap to the information visualization field.

### 2.1. The many roads to Rome

**Fig. 4:**
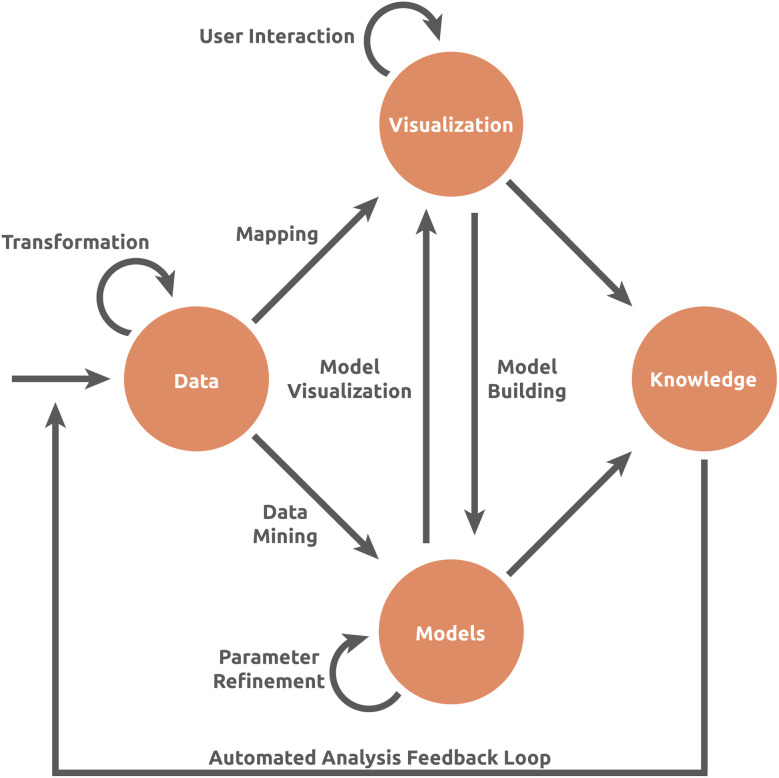
The visual analytics pipeline of Keim *et al.*^26^

Untargeted metabolomics and its data analyses challenges are exploratory in nature. It is hence worth considering, how a researcher, given some data and some (interactive) visualization system, reaches conclusions, formulates and validates hypotheses, or generates new knowledge about their data. The study of these processes is at the core of the field of visual analysis and goes beyond information visualization itself.^24^ Different visual analysis models have been proposed to describe the process of gaining insight from data through visual analysis.^25^ These models usually represent sensemaking as a non-linear, often (semi-)circular process involving data, models, visualizations, and knowledge, all connected by user-driven interaction (Fig. 4).^26^ Some of these models attempt to break down this process further, by either conceptualizing “Hypotheses”’ and “Domain knowledge” as separate entities,^27^ or conceptualizing such visual analytics models as a dialogue between the user and the visualization tool.^6^ While the details of such models may be theoretical and abstract, appreciating the potential interplay between users, data, the visual system, and knowledge can assist developers in designing novel visual systems.

### 2.2. Towards targeted tasks

**Fig. 5:**
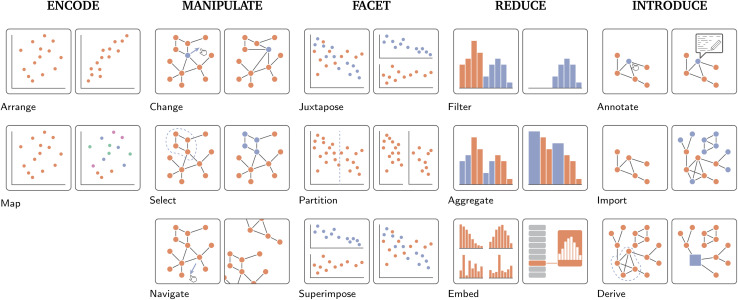
Brehmer and Munzner's^2,39^ visualization “How's” of designing visual idioms, *i.e.*, “Encode”, “Manipulate”, “Facet”, “Reduce”, and “Introduce”, each of which features two to three lower-level approaches thereto. Encode describes how data is visually represented and presented to the user. Its two constituent approaches, *i.e.*, “arrange” and “map”, are simply two examples of a much larger, exhaustive set discussed by Munzner.^2^ Manipulate describes different approaches to interactively altering the visual representation, such as, within the context of network visualization, “changing” the layout of a graph by clicking and dragging nodes, “selecting” groups of nodes to highlight them, or “navigating” through a visualization through zooming and panning. Facet describes the act of viewing different aspects of the same dataset or viewing the same dataset in a different way. “Juxtapositioning”, for example, is the process of breaking one view into multiple views based on some categorical variable. “Partitioning” describes breaking up one view into multiple based on some (user-defined) point along some variable. Lastly, “superimposition” is the opposite of juxtapositioning, *i.e.*, bringing two separate views of the data together into a single one. Reduce is simply the process of reducing the visual complexity presented. In its simplest form, this takes the form of “filtering”, *i.e.*, the removal of data not currently of interest. “Aggregation” is the creation of visual summaries of data points, *e.g.*, the grouping of two or more bars of a bar chart into a single bar. More complex, “embedding” describes summarizing all data points into some form of visual abstraction, *e.g.* individual bar charts into a list of glyphs. Finally, Introduce is the introduction of new visual elements or data to an existing visualization, such as “annotating” a node's attributes in a node-link diagram, “importing” another (sub)graph alongside some already embedded one, or “deriving” a new data element based on existing data.

**Fig. 6:**
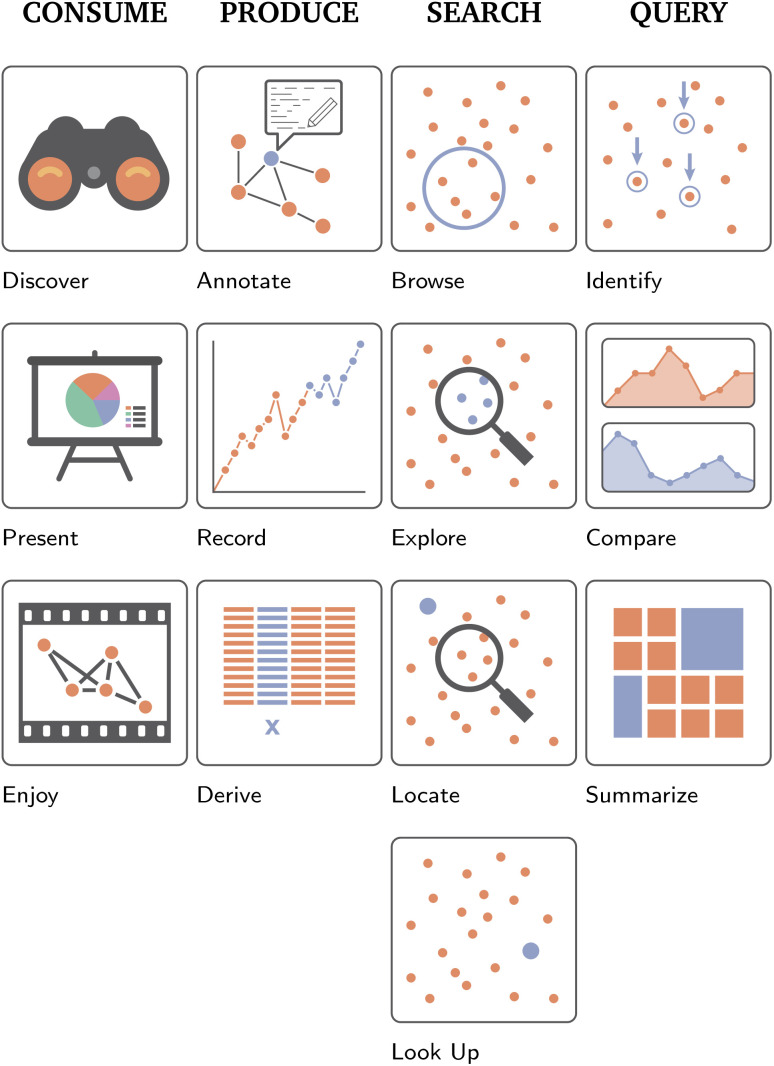
Brehmer and Munzner's^2,39^ visualization “Why's” underlying the use of visualization, *i.e.*, why a particular task is performed. Here, the authors identify four broader goals, comprised of higher levels ones, *i.e.*, “Consume” and “Produce”, mid-level goals, *i.e.*, “Search”, and low-level goals, *i.e.*, “Query”. Each of these bigger-picture goals is additionally broken down further into three to four categories. First, consume describes the common uses of visualizations by domain experts and lay people. This includes “discovering” novel aspects about their data, such as validating or generating novel hypotheses, “presenting” data in a targeted manner to others, or merely “enjoying” a visual representation casually. Produce is the creation of novel “artifacts”, such as adding “annotations” to a dataset, “recording” new (visual) data, such as an ongoing time-dependent process, or “deriving”. Searching, as the name implies, is the process of locating particular data of interest. Depending on whether the data itself and its location in the visualization is known *a priori*, this process is either described as “exploring” (both target and location unknown), “browsing” (location known, target unknown), “locating”, (location unknown, but target known) or “looking up” (both target and location known). Finally, query describes the low-level processes a user aims to complete once their target(s) have been located; “identification” is the process of returning characteristics of particular data, “comparison” is the visual comparison of data characteristics, and “summarization” is the process of aggregating characteristics of the target data.

The goals of information visualization can be dichotomized into (i) the (interactive) exploration and analysis of data, and (ii) the communication of findings from (visual) data analyses.^23^§§Visualizations designed to be consumed by or presented to users to communicate a particular set of findings are sometimes called “Infographics”. While conventionally made by hand, members of the information visualization community have made efforts to both assist this manual generation process^28,29^ and to partially automate their generation.^30,31^ These very different goals each come with their own types and metrics of evaluation. On the one hand, visualizations designed to assist experts in their exploratory analyses strive to facilitate insight generation.^32^ On the other hand, visualizations enabling the communication of results, strive to improve elements such as memorability, engagement, or aesthetics.^33–35^ In addition to the coarse dichotomization of goals, several task taxonomies at different levels of granularity have been proposed over the years, some trying to classify visualization goals in comprehensive and abstract fashion, others attempting to explore the goals of domain/data-specific visualizations. These task taxonomies all attempt to describe the interplay between the data itself, the users, and the tasks^8^ in order to guide the design, and possibly validation, of visualization systems.^36^ A core mantra often adhered to in the design of information visualization is Shneiderman's “Overview first, zoom and filter, then details-on-demand”^7^ or van Ham and Perer's “Search, show context, expand on demand”.^37^ However, domain-specific opposites to these manta's also exist.^38^ While many possible taxonomies would warrant discussion here, we opt to discuss that of Brehmer and Munzner^2,39^ owing to its popularity and conceptual simplicity. In order to disentangle the abstract goals that inform particular tasks from the low-level tasks themselves, they distinguish between the “Why”, the “What”, and the “How”. Intuitively, “How” describes the low-level tasks and means through which the user's goals are achieved (Fig. 5), the “What” describes the inputs and outputs of the visualization system, and the “Why” describes the larger goals of the user (Fig. 6). The low-level How tasks that users must engage with to accomplish their goals can be categorized in a myriad of different and usually domain-specific ways. We refer the interested reader to the domain-agnostic categorizations in the form of Shneiderman *et al.*‘s^7^ aforementioned discussion of general tasks in information visualization and Amar *et al.*'s^40^ low-level task taxonomy for information visualization. Here, metabolomics tool developers can benefit from these existing taxonomies, as these, while very general, should allow them to better understand and appreciate the possible needs of their user group. However, we also see ample opportunity for metabolomics experts to create taxonomic classifications of their own in order to better communicate their domain-specific tasks to developers and visualization experts.

### 2.3. The many methods of mapping meaning

**Fig. 7:**
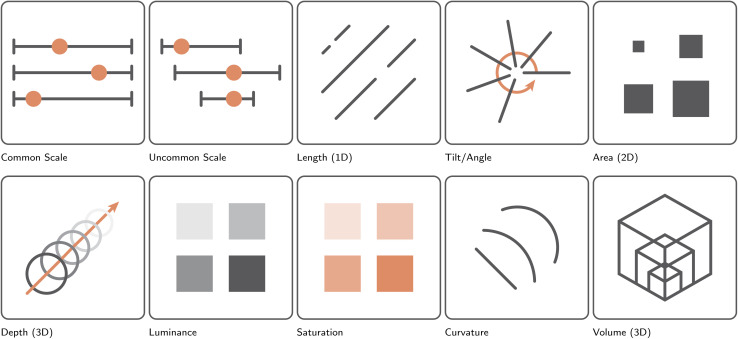
Presentation of the effectiveness of different visual channels for communicating variable magnitude, ordered according to Munzner's effectiveness ranking from left to right, row by row.^2^ More specifically, in order of effectiveness: positions on a common scale, positions on an uncommon scale, angle, 2D length, 2D area, 3D depth, color luminance, color saturation, 2D curvature, and 3D volume.

**Fig. 8:**
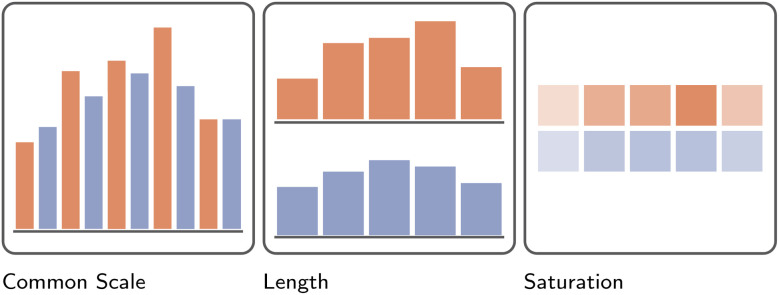
Illustration of the effectiveness of different visual channels to communicate magnitude.^42^ Here, across five hypothetical points in time, the magnitude of an imaginary variable has been recorded for two separate groups, colored orange and blue, respectively. In accordance with the ranking of visual channels presented in Fig. 7, the differences between these groups, as well as the overall trend of the data, is understood best when presented as positions on a common scale. Less effective is reliance on each bar's length to communicate differences. The least effective, at least for the example presented here, is the use of color saturation.

**Fig. 9:**
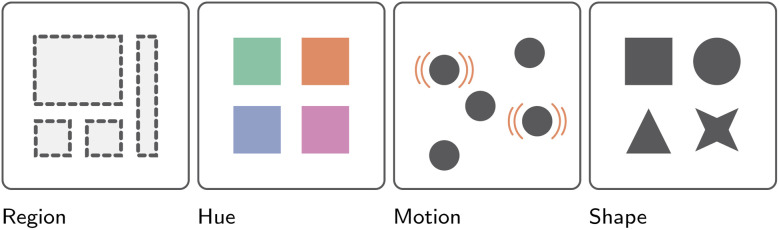
Presentation of the effectiveness of different visual channels for communicating variable identity, ranked from left to right.^2^ More specifically, from most to least effective: region, color hue, motion, and finally shape.

So far, the discussion of information visualization has been purposefully abstract, *i.e.*, discussions of goals, tasks, and sensemaking models. Here, we discuss how to communicate data and its properties visually. A variety of so-called visual channels are used to do so, including color, shape, angle, position, and others (Fig. 7–9).^2^ However, not all visual channels are created equally, some having been found to be more or less effective than others.^41^ Developers must therefore carefully consider which channel to use, depending on the importance of the data relative to other data presented, the scale of the data, its absolute magnitude, *etc*. Consider, for example, a simple bar chart representing the magnitude of two categories (A and B) across time, compared to two juxtaposed bar charts and a color saturation plot, all three visualizing the same data (Fig. 8). While at first, all visualizations superficially appear interchangeable, closer inspection, in line with Munzner's own ranking of such channels (Fig. 7), reveals the common scale approach to be the most effective in allowing readers to visually compare relative magnitudes. By utilizing the “Position on a common scale” channel, a reader can much easier compare differences between categories at the same time point, but also observe trends across time.^42^ Making use of theoretical considerations, and more importantly, empirical user studies, the visualization community has made concerted efforts:

• to understand the relative importance of the different visual channels (Fig. 7 and 9),^41,42^

• to understand the effectiveness of individual channels for specific purposes, such as the use of animation to communicate dynamic trends,^43^

• to provide guidelines on how to effectively use visualization channels, such as how to effectively use color in visualizations,^44^ how to best encode edge weight in adjacency matrices,^45^ or how to best evaluate the quality of visualization across information visualization (sub)domains,^46^ and

• to outline and characterize common pitfalls, such as the use of so-called “rainbow colormaps” frequently and erroneously employed by domain experts and developers alike^47,48^ or the still common usage of color-blindness unfriendly red-green color mapping across domains.^49,50^

### 2.4. Evaluating efficacy effectively

Information visualization guidelines, recommendations, and warnings are not conjured from thin air, but derived from empirical user studies. Commonly, study participants are tasked with completing a series of (low-level) analyses or answering several questions using multiple visualization approaches in order to evaluate participant performance differences between the different visualizations.^10^ Additionally, participants' subjective experiences may be recorded. Results are frequently analyzed either quantitatively or qualitatively, and less commonly in mixed methods studies. Unsurprisingly, given the increasing importance of such evaluations in information visualization,^51^ many review papers and state-of-the-art reports have been published to, in turn, provide a list of guidelines,^52,53^ best practices,^54^ and common pitfalls^32^ when conducting different types^55^ of user studies.^52,56^ When evaluating the efficacy of a visual system, a historically often neglected challenge only truly tackled in recent years,^57^ is the need for visualizations to remain accessible to the visually impaired or even blind^58^ through, for example, sensory substitution.^59^ While such concerns may initially sound alien and strange to the metabolomics community, it is worth considering that visual impairments, such as colorblindness, affect 8% and 0.5% of the male and female population, respectively.^49^ Here, an easy step to ensure one's visualizations remain accessible is the simple use of colorblind-friendly colormaps and scales.^50^ Additionally, user studies can bring rigor to the study and evaluation of visualization approaches and systems. While not formally used in the metabolomics field, they would be a worthwhile addition to the tool development and tool review process. More specifically, we see an opportunity for the developers of interactive (untargeted) metabolomics visualization platforms to go beyond visual case studies or proof-of-principles in order to more rigorously evaluate the efficacy of their software's interactive visualization component, inspired by the work of the information visualization community.

### 2.5. Unraveling complexity with interactivity

So far, our primer on information visualization has squarely side-stepped the critical role and importance of user interaction in information visualization.^60^ Several taxonomies have been put forth to capture and describe possible modes of interaction between user and visual system.^7,61,62^ Interaction techniques can allow for visualization to be more engaging, create richer visualizations with additional on-demand information, or facilitate interactive data exploration and analysis. Crucial for metabolomics research, interaction is the primary gateway to dealing with large and complex data in exploratory data analyses. Conventional mouse and keyboard-based interactive approaches providing adjustable filtering steps, on-demand information displays, and connection of different streams of information through dashboard interfaces play an exceedingly important role in data integration and visualization, especially in the era of large data. Going beyond 2D representations on a computer screen, cutting-edge experimental visualization technologies are moving beyond these established frameworks towards the inclusion of 3D augmented reality and virtual reality applications which bring new possibilities of interaction with them.^63^ Here, metabolomics researchers and developers may be inspired by the kind of work done in VR for immersive protein structure visualization^64–66^ or immersive large network visualization.^67–69^ For example, one could visualize mass spectral and structure embeddings to obtain an overview of the chemistry present in their metabolomics data, with 3D interactive visualizations allowing users to select and interact with specific types of chemistry. Such interaction with the data could enhance understanding and could be beneficial to teach concepts like chemical space. However, while 2D or 3D interaction techniques can be critical to creating effective visualizations, their potential “costs” should also be carefully considered.^70^

### 2.6. Information visualization meets metabolomics

In this section, we merely provided a bird's eye view of the complex and multifaceted domain of information visualization, as each of the discussed facets is an entire field of study unto itself. Ultimately, we wish to highlight that the creation of effective visualizations is not merely an aesthetics-driven endeavor, but a quantitative science and active field of research. While the referenced papers, reports, surveys, and reviews should not be viewed as definitive,^51^ we do believe they make clear the necessity for metabolomics experts and developers alike to think more critically about how they choose to visualize their data and why. To those looking to go beyond this primer, we recommend reading the works of Munzner,^2^ Camm *et al.*,^71^ and Midway^72^ for deeper, but still approachable, discussions of information visualization. They may further profit from digging deeper into subdomains of interest to them. Beyond encouraging readers to immerse themselves in the field of information visualization, however, we see a great opportunity for collaboration between metabolomics and visualization experts. Domains, such as metabolomics, provide interesting data, challenges, conventions, and analytical goals that could prove inspirational to the greater visualization community. On the flip side, visualization experts bring experience and expertise to assist in the design of better visualizations and visual systems for those same domain experts.

## 3. Network visualization – a primer

Complex relationships between entities are commonly expressed mathematically as a graph.^73^ Over the past decade, graphs have found application across many domains, from social networking,^74^ where entities represent people connected by different types of relationships, through to more abstract Bayesian network structure learning endeavors, in which entities represent random variables of the Bayesian model and the relationships statistical dependencies between them.^75^ They have also found application in metabolomics *via* metabolic networking,^16^ where entities represent metabolites connected by reactions, or molecular networking,^76^ where entities represent mass spectral features connected by spectral similarity. Graph drawing, sometimes interchangeably referred to as network visualization, is the science of visually displaying network graphs. Graphs are made of nodes and edges, but, in most cases, these nodes do not possess an intrinsic position in either two-dimensional (2D) or three-dimensional (3D) space. Therefore, a central aspect of network visualization is the development of layout algorithms to position nodes. Additionally, network visualization deals with the empirical evaluation of layout and visualization efficacy, and the development of novel visualization approaches for particular domain-specific applications. Here, in light of the importance of network visualization in the field of untargeted metabolomics *via* tools such as Feature-based molecular networking^77^ and beyond,^78,79^ we aim to provide a gentle introduction to network visualization, *i.e.*, by discussing the most common layout approaches, different types of evaluation strategies with which to measure a layout's quality, and providing guidance on when to use which network representation.

### 3.1. Just a formality

In its simplest form, a graph is described as a tuple *G* = (*V*, *E*), consisting of a set of entities, also known as nodes or vertices, *V* = {*v*_1_, *v*_2_, …, *v*_*n*_} and a set of, here undirected, relationships, also known as edges or links, *E* = {*e*_1_, *e*_2_, …, *e*_*m*_} ⊆ *V* × *V* connecting them, where *n* = |*V*| and *m* = |*E*|.^80^ More complex graph models exist, such as hypergraphs, *k*-partite graphs, clustered graphs, or multilayer graphs.^81^ In addition, nodes, as well as edges, may potentially be enriched by (multivariate) attributes. However, in the interest of simplicity, we limit ourselves to the above common and simple definition. Here, it is important to note that a graph is an abstract data type without intrinsic visual representation. Thus, at the core of any network visualization endeavor lies some abstract graph drawing algorithm to convert a graph *G* = (*V*, *E*) to a geometric representation. Most commonly, graphs are represented as node-link diagrams, commonly referred to as just networks in metabolomics. In node-link diagrams, nodes *v* ∈ *V* are mapped to (labeled) points, circles, rectangles, or glyphs (*i.e.*, a visual representation of a piece of data) placed in a 2D space at some position *p*_*v*_ = (*x*_*v*_, *y*_*v*_), and edges *e* ∈ *E* are represented by straight (or curved) line segments connecting the two endpoints *p*_*v*_ and *p*_*u*_.^73^ While many more complex representations exist, we limit our discussion below to the four most common node-link diagrams as well as two prevalent alternatives, namely adjacency matrices and hybrid visualization approaches.

### 3.2. Give it to me straight

**Fig. 10:**
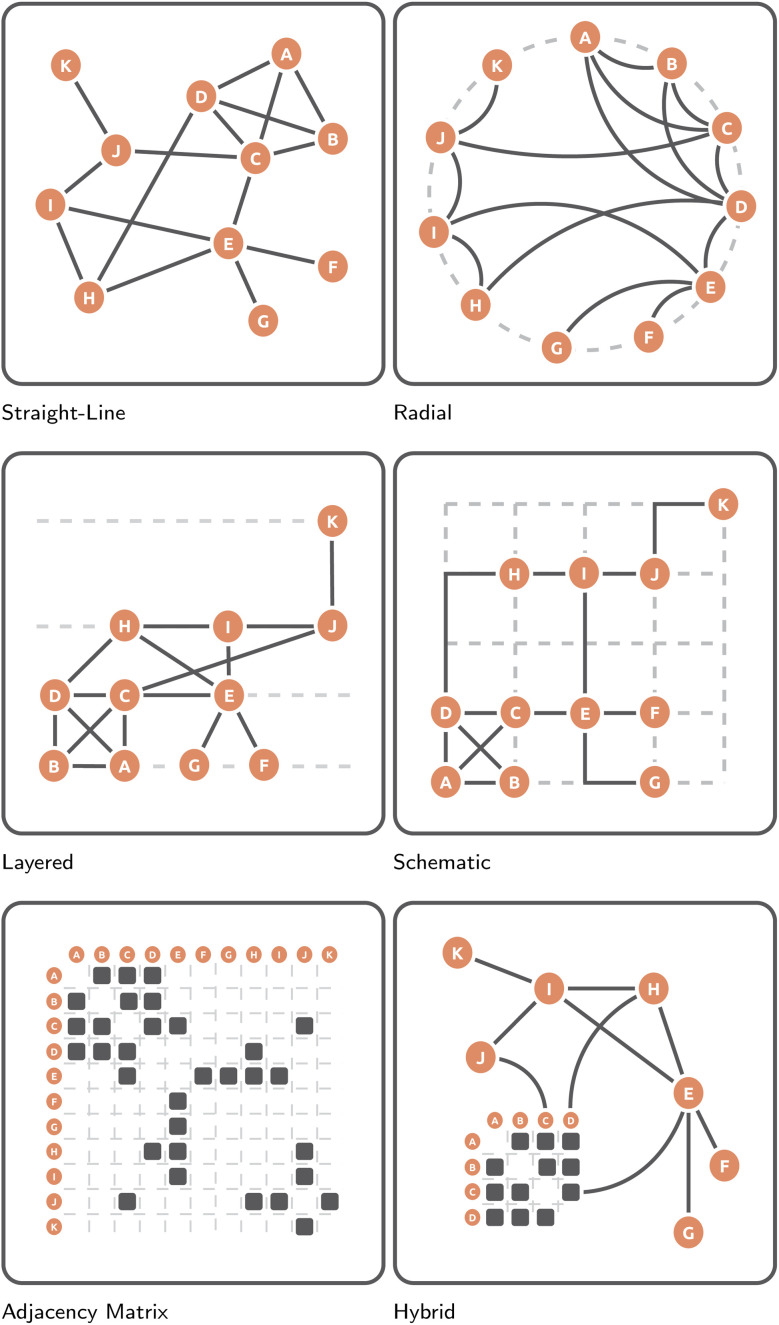
Illustrative representations of the here-discussed abstract graph layout approaches, *i.e.* straight-line node-link diagrams, radial node-link diagrams, layered node-link diagrams, schematic node-link diagrams, adjacency matrices, and hybrid approaches. All six representations utilize the same graph *G* = (*V*, *E*), where *V* = {*A*, *B*, *C*, *D*, *E*, *F*, *G*, *H*, *I*, *J*, *K*}, with the same |*E*| = 16 undirected edges between them.

Straight-line node-link diagrams are by far the most common and popular visual network representation. As the name suggests, a graph's nodes, represented commonly as labeled circles, are connected by straight-line segments (Fig. 10 (Straight-line)). Intuitively, nodes that are connected by edges should be placed close together to each other in 2D space, whereas those that are not should be placed further apart. Building upon this intuition, many algorithms for the layout of such node-link diagrams have been developed over the years. Arguably the most commonly utilized family of such algorithms are so-called spring-embedding and force-directed algorithms. These algorithms model graphs as a physical system that exerts attractive and repulsive forces upon its nodes.^82^ The placement of nodes is then iteratively refined as a function of the system's forces. The three most common spring embedders are (extensions of) the Eades,^83^ Fruchterman–Reingold,^84^ and Kamada–Kawai algorithms.^85^ Eades^83^ is arguably the most conceptually simple approach to spring-embedding graphs: nodes are modeled as magnetized balls and edges as springs connecting them. For some random initial placement of nodes, the algorithm iteratively calculates the forces between two connected vertices as a function of the Euclidean distance between them and the spring's modeled rest state. Moreover, non-adjacent nodes “magnetically” repel each other as a function of the Euclidean distance between them. Building upon this seminal work of Peter Eades, Fruchterman and Reingold^84^ model vertices as “atomic particles” that exert attractive and repulsive forces upon each other to achieve a more even node distribution in 2D space. Additionally, the algorithm adds “temperature” to the force simulation, which starts at some maximum and decays towards zero. The higher the temperature, the larger the movement nodes admitted by the algorithm. This special form of simulated annealing ensures that, as the layout iteratively improves, the adjustments made to node placement become smaller and smaller. Finally, taking a very different approach, Kamada and Kawai^85^ redefine the notion of “quality” in the produced graph layout: nodes' Euclidean distances to each other are now a function of their graph theoretic distance, *i.e.*, all-pairwise-shortest-paths. For computational reasons, a spring model is used as well, *i.e.*, nodes attract or repel each other if their geometric distance is larger or smaller than their graph theoretic distance, respectively. No matter how they are laid out, straight-line node-link diagram representations have found frequent use across many different domains, most likely owing to their common availability in many libraries and software packages as well as their conceptual and visual simplicity: they are intuitive to read and many people have a degree of *a priori* familiarity with this representation. On a computational level, they are easy to implement and scalable. This makes them a broadly applicable family of network visualizations, which has resulted in them finding wide use in applications visualizing gene regulatory networks,^86^ protein–protein interaction networks,^87^ multi-omics networks,^88^ social networks,^89^ ego networks, or compound graphs,^90^ among many others.^91^ However, spring embedded or force-directed straight-line node-link diagrams are not without their drawbacks. Crucially, these heuristic algorithms do not bring with them any formal guarantees regarding the produced drawing quality, owing to the many local optima underlying the physical model.^92^ Moreover, they only indirectly optimize graph aesthetic metrics¶¶Given some input graph *G* = (*V*, *E*) and some abstract graph drawing algorithm, the question arises of how “good” of a drawing has been produced. Earlier graph drawing research on the quality of algorithmically laid out network visualizations focused on so-called graph aesthetic metrics.^93^ Simply put, these metrics aim to quantify the quality of graph drawing based on its placement of nodes and edges. Examples hereof include the number of edge crossings, edge crossing angles, minimum edge angle ratios, and node orthogonality. Importantly, however, such aesthetic metrics, on their own, do not describe how readable a particular graph drawing is to a human user. Subsequently, efforts were made to empirically study the effect of these metrics on human understanding of graphs.^94^ Here, while all these metrics are important to some degree, the key aesthetic metric with maximal impact on readability is the edge crossing number,^95^ even for larger graphs.^92^ Research efforts were subsequently made to minimize or resolve edge crossings in different ways, such as splitting vertices^96^ or representing node-link diagrams in 3D.^97^ of importance,^92^ and, especially for larger graphs, frequently produce cluttered and unreadable drawings, often referred to as “hairballs”.^98^ It is probably because of these drawbacks, that straight-line node-link diagrams are frequently employed alongside other graph representations, such as orthogonal/schematic representations^99^ or radial node-link diagrams.^100^

### 3.3. Circular logic

Radial node-link diagrams are a more visually constrained form of the aforementioned straight-line node-link diagram. More specifically, instead of allowing nodes to be drawn freely in 2D space, their placement is restricted to the circumference of some given circle (Fig. 10 (Radial)). Edges are commonly represented as arcs within the confines of this circle. The exterior of the circle is typically used not for topological information, but for some form of additional information, such as node labels, node attributes, or summary statistics. These representations often aim to produce a uniform distribution of nodes along the circle. However, node placement can also be a function of the number of induced edge crossings or the (hierarchical) group structure of the nodes themselves. A popular framework for the layout of radial node-link diagrams, especially in biological/biochemical application areas, is the Circos library^101^ or the force-directed (particle-based) D3 system.^102^ Beyond just their aesthetic appeal, a key advantage of radial graph drawings is the ability to easily visualize (disjoint and hierarchical) grouping for the one-dimensional (1D)-ordered entities along the circle's circumference. Examples hereof can be found in spectrometry result visualization^99^ or genetic pathway visualization.^103^ Additionally, nodes can be placed along different concentric rings to visually communicate additional group structures, such as the function modules of miRNA,^104^ different alter-levels in ego networks,^105^ different subgraphs of a larger graph,^106^ or individual time-slices of a larger graph.^107^ However, a key drawback of radial drawings is their poor scalability with increasing nodes and edges, in part owing to their inefficient use of space.^108^ Here, (hierarchical) edge bundling is frequently employed as a strategy to overcome the visual clutter introduced by too many edges.^109,110^ In addition, node aggregation, *i.e.*, hypernodes, is an approach frequently employed to reduce node clutter.^111^

### 3.4. Peeling back the layers

Similar to the previously discussed radial node-link diagrams, layered node-link diagrams restrict the placement of nodes. More specifically, instead of allowing nodes to be placed freely in 2D space or forcing them along the circumference of a circle, layered node-link diagrams restrict node placement to parallel, equidistant lines (Fig. 10 (Layered)). The two key degrees of freedom of such representations are the distribution of vertices among layers and the ordering of vertices within a layer. Edges are commonly represented as straight-line segments. For the layout of these graphs, several heuristic approximations^112,113^ to the computationally demanding steps of the popular Sugiyama framework^114^ have been put forth. Most commonly, such layered representations are used to visualize tree graphs, such as phylogenetic trees.^115,116^ Examples of their use can also be found in the context of ego network visualization across domains.^117^ However, actual examples of these layered graph representations in the wild appear to be few and far between. Their key limitation is the highly restrictive visual representation that make them suitable to only tree(-like) graph representations. Moreover, they also appear to be more difficult to layout, both because of the computational demands of the algorithm, and the lack of readily available implementations in software packages and libraries.

### 3.5. The grid: a digital frontier

Especially for larger and more complex graphs, a straight-line node-link diagram may produce graph drawings that are too cluttered and subsequently hard to read.^82,92^ In such situations, a schematic network representation can be employed instead. Here, nodes are placed on a 2D grid, and edges are drawn as polylines with a restricted set of slopes.^118^ Such slopes may be rectilinear (Fig. 10 (Schematic)) or octolinear,^119^ akin to public transportation or city maps.^118^ When drawing node-link diagrams in such a manner, a number of visual aesthetic criteria are typically optimized, such as minimizing the grid size, minimizing edge bends, or minimizing the number of edge crossings.^11^ Within the context of biochemical network visualization, schematic node-link diagrams make up the second largest group of visualization approaches, most likely owing to domain experts' familiarity with certain canonized (and often hand-drawn) schematic layouts, whose layout style is subsequently often reproduced to appeal to those experts, as seen in tools designed for the visualization of KEGG^120^ or Reactome pathways.^121^ Alternatively, the metro-map metaphor has also been embraced to visualize synaptic connectivity^122^ and metabolic pathways.^11^ A key advantage of such schematic layouts is their visual appeal as well as their visual simplification of a graph's topology, which has been hypothesized to make them more intuitive and engaging to use.^123,124^ However, similar to the previously discussed layered node-link diagrams, a key limitation of schematic representations is the computational cost of laying them out; a possible explanation for the many hand-drawn schematic layouts out there.^124^

### 3.6. Enter the matrix

Unlike all previously discussed node-link diagram-based representations, adjacency matrices do not visualize nodes as (labeled) circles or rectangles and edges as line segments but instead opt to visualize a graph in tabula fashion (Fig. 10). More specifically, for some undirected, non-weighted graph *G* = (*V*, *E*), an adjacency matrix takes the form of an |*V*| × |*V*| boolean matrix, where each vertex *v* ∈ *V* is represented twice, once as a column and once as a row. If an edge {*v*_*i*_, *v*_*j*_} ∈ *E* exists between nodes *v*_*i*_, *x*_*j*_ ∈ *V*, then the matrix cells (*i*, *j*) and (*j*, *i*) take on the value 1. Otherwise said cells take on the value 0. Visually, matrix cells of value 1 are often “filled-in” or “colored-in” (Fig. 10 (Adjacency matrix)). This tabular representation brings with it some important advantages. First and foremost, the previously discussed graph aesthetic metrics simply do not apply: there can be no edge crossings, edge/node occlusions, or minimum edge angle ratios. Subsequently, it has been argued, and partially demonstrated, that adjacency matrices scale better (in terms of their usability) with larger and more complex graphs than node-link diagrams.^125–127^ Second, comparable to radial node-link diagrams, the 1D arrangement of nodes along an adjacency matrix's rows and columns, coupled with their uncomplicated reordering, straightforwardly allows for the visual highlighting of different patterns of connectivity, *e.g.* different node clustering can highlight the connectivity within and without certain subgraphs.^128,129^ As will be discussed later, these conceptual advantages have resulted in some researchers recommending adjacency matrices over (straight-line) node-link diagrams for particular low-level graph analysis tasks, such as counting edges or finding nodes.^125,127^ Adjacency matrices have found use in biochemical network visualization, especially in visual gene (co-) expression analysis,^130–132^ where paired with canonical orthogonal/schematic representations from the domain, they are frequently used to visualize the derived correlation or co-expression network as a clustered heatmap matrix. However, adjacency matrices are not without their downsides. First, it is often hypothesized that many users are *a priori* less familiar with adjacency matrices than node-link diagrams,^45,133^ which may necessitate longer and more in-depth training.^134,135^ Second, the results of the last 20 years of comparisons between adjacency matrices and node-link diagrams sometimes fail to paint a favorable picture for matrices, both in terms of user performance or experience, as well as certain types of tasks in particular, *e.g.* shortest path-finding tasks.^126,136,137^ Lastly, the sensitivity of adjacency matrices to their node ordering can also be seen as a weakness, in that different ordering must be made available to a user to ensure they can fully understand different relationships within the data.^129^

### 3.7. Curious crossbreeds

A final and difficult-to-categorize group of network visualizations are so-called hybrid approaches, which are some of the aforementioned styles of network visualizations merged and augmented into something new. Two examples of such hybrid network visualization techniques are Henry *et al.*‘s NodeTrix^138^ and Angori *et al.*‘s ChordLink.^139^ Here, Fig. 10 (Hybrid) represents a hybrid representation akin to NodeTrix, *i.e.*, a combination of an adjacency matrix and node-link diagram. More specifically, the larger graph to be visualized is broken down into individual, dense subgraphs, each of which is then visualized as a separate adjacency matrix to reduce visual clutter. Connections between these subgraphs' adjacency matrices are then visualized as line segments, akin to a node-link diagram. Similar to NodeTrix, ChordLink breaks the larger graph down into smaller and denser subgraphs. However, instead of visualizing these as adjacency matrices, they are visualized using a type of radial node-link diagrams, referred to as chord diagrams. Here, connections between these subgraphs are then visualized as straight-line segments. Unsurprisingly, given their conceptual and implementation complexity, such hybrid approaches are seldom found in practice. NodeTrix, for example, was utilized to visualize human brain connectivity, where each adjacency matrix represented a pre-determined component of the human brain.^140^ Given the broad nature of this category of network visualization approaches, it is effectively impossible to speak about their universal advantages and disadvantages.

### 3.8. Tackling tough tasks

With many different network visualization approaches presenting themselves, the question naturally arises of which one to use. To answer this question, one must first consider what kind of (low-level) graph analytical tasks users of said visualizations are expected to complete. Once one has a grasp of these tasks, one can, as previously discussed (Section InfoVis), quantitatively and qualitatively probe the performance and experience of users for (a subselection of) these tasks. Here, several efforts have been made to identify and taxonomically classify the kind of graph analytical tasks users complete. Arguably the most well-known and applicable one of these taxonomies, is Lee *et al.*‘s^141^ low-level graph task taxonomy. However, more specialized graph task taxonomies exist as well, such as Saket *et al.*'s^142^ taxonomy for group-level tasks in graphs. For dynamic graphs specifically, several taxonomies have been put forth over the years.^143–145^ Lastly, and maybe most interestingly to metabolomics domain expert readers, Murray *et al.*^146^ developed a graph task taxonomy for the analysis of biological pathway data specifically. Here, we base our discussion on the aforementioned Lee *et al.* taxonomy^141^ owing to its widespread use and applicability. This taxonomy breaks graph analysis tasks down into low-level tasks, such as filtering, sorting, clustering, *etc.*, and complex graph tasks, which are the focus of this discussion. These complex graph tasks are comprised of four classes, namely (i) topological, (ii) attribute-based, (iii) browsing, and (iv) overview tasks. Each of these classes is then further broken down into low graph analysis level tasks.

It is these low-level tasks that are commonly the focus of, especially quantitative, user studies. For a selection of these tasks, participants are challenged to complete them on a hitherto unseen graph using one or multiple network representations, depending on the within/between-subject nature of the study. Following Ghoniem *et al.*‘s^125,147^ definition of “readability”, the more “readable” a representation, the faster and more accurately a user should be able to complete such tasks. Thus, studies tend to track both the response time and answer accuracy to compare them across experimental conditions, here network representation, though graph size and complexity are also commonly studied. Here, it is interesting how, despite their popularity and many types, node-link diagrams have not been studied and compared as systematically as one might imagine. While, graph aesthetic metric comparisons exist, task-driven and quantitative user studies are much less common. Moreover, the few comparisons that do exist yield inconclusive results, conceding that, while node-link diagram representation has a (statistically significant) impact on user performance, no pairwise differences between representations can be detected.^148–150^ However, for completeness, it should be mentioned that Didimo *et al.*‘s^137^ found that orthogonal (schematic) representations were superior to the other node-link diagrammatic representations investigated, at least for overloaded graphs. Rather than comparing different node-link diagram representations, the attention of the visualization community has largely been on the comparisons of node-link diagrams with adjacency matrix representations. Starting with the seminal studies of Ghoniem *et al.*^125,147^ twenty years ago, several studies have followed up upon, refined, and refuted the findings of the original authors. It would thus be fair to say the field remains conflicted. For a number of low-level tasks, such as adjacency tasks, accessibility tasks, and overview tasks, results both in favor^127,151,152^ and against^153,154^ adjacency matrices have been produced. However, there do exist some tasks, such as common connection tasks and shortest path finding/tracing, for which the superiority of node-link diagrams over adjacency matrices has been by now well documented.^126,134,136,137^

In such comparisons, some key points are repeatedly brought up. First, it is common for authors to hypothesize that users are *a priori* more familiar with node-link diagrammatic representations than adjacency matrices, which can have a notable impact on user performance.^45,136,155^ In line with this, it argued and partially shown, that adjacency matrices require additional effort and time to be learned and grasped by users unfamiliar with the representation.^134,135^ This points towards the need for careful consideration of when and how to employ adjacency matrices. On a slightly more technical level, adjacency matrices have been argued to scale better with increasing nodes and edges, owing to the complete absence of node and edge occlusions thanks to its tabular representations.^126,127^ This tabular presentation of graph topology has also been argued to make overview and node-lookup tasks as users need only parse a 1D ordering of nodes as opposed to a 2D smattering of them.^125,154^ Similarly, the orderly arrangement of edges has been argued to make edge-counting and adjacency tasks easier as well,^126,136^ though results are far from conclusive.^127,137,153^ The tabular setup, however, also appears to make common neighbor tasks and shortest path finding/tracing tasks significantly more difficult.^126,134,136,137^ Here, instead of being able to simply follow a series of lines/arcs from one node to another to either follow a path or check common neighborhoods, matrix users must check rows and columns against each other iteratively for such tasks. All in all, it should be stressed that there is no singular, one-size-fits-all, “best” network representation. Instead, depending on the data, the experience and expectations of the user group, and the tasks they are going to complete, different network representations offer themselves up. However, provided a user group is not particularly familiar with any network representation and will not receive extensive training on the use of a particular representation, it is likely best to utilize some straight-forward straight-line node-link diagram.

### 3.9. Concluding comments

In this section we have outlined the importance of graphs to various analysis endeavors across domains, explored the basics of abstract graph drawing algorithms, and discussed various network representations both in terms of their produced graph aesthetic metrics, as well as their impact on user performance and preference. Here, while no singular “best” representation can be identified across domains and use cases, we hope to have outlined the impact that these representations can have on users. Ultimately, we have intended to highlight approaches to graph drawing that go beyond the straight-forward (spring embedded/force-directed) node-link diagram to point both metabolomics experts and developers to additional tools for visually communicating the topology of their networks. The many practical use cases of network visualization within the context of untargeted metabolomics are explored in more detail in the User roadmap (Section 4).

## 4. User roadmap

In this section, we provide a highlights overview for each analysis stage of the untargeted metabolomics workflow with respect to tools and their visualization capabilities. We provide an assessment of the utility, as well as strengths and weaknesses, of the visualizations highlighted. Moreover, we discuss which visualization strategies might be worth including in future endeavors. In so doing, we provide the reader with an overview of the possibilities and available capabilities within the untargeted metabolomics data analysis workflow. A common theme among the tools discussed will be the implementation type, as implementation often comes in different degrees of automatization, accessibility, customizability, and reproducibility. Here, we note that visualizations are usually components of larger tools, sharing a combined user interface. We roughly delineate between three main categories, namely completely automated graphical-user-interface-based (GUI-based) tool sets, pre-defined script-based tools, and custom manual scripts or custom GUI-based workflows. Completely automated GUI-based tools usually provide extensive support and accessibility for researchers with no coding background, allowing them to quickly produce visualizations for their data. They are, however, often more limited in their customizability and require extensive developer maintenance work. For such tools to be part of reproducible workflows it is important to have good tracking of the settings used. Here, GNPS, MetaboAnalyst, and MZmine are excellent examples of GUI tool sets with setting transparency.^156–158^ Pre-defined script-based workflows offer similar levels of streamlining to the first, yet provide more customizability and extendability, at the cost of requiring some scripting knowledge. These workflows are build by developers and can be used and extended by researchers with scripting skills. Here, it is relatively straightforward to inject additional processing steps or modify the data that is passed to visualizations. When using interactive notebooks, *e.g.*, based on Jupyter or RMarkdown, reproducibility can be ensured even for highly customized workflows. Examples of such workflows are tools like MolNetEnhancer, the MetaboAnalyst R package, UmetaFlow, and components of the XCMS ecosystem.^159–162^ Finally, custom scripting, as well as the custom creation or customization of existing GUI-based visualization frameworks, tends to be the most laborious and time-consuming, requiring extensive familiarity with the data and visual analysis approach, as well as scripting skills or deep familiarity with the GUI tool set(s) used. While highly flexible, this will usually only be used by expert users, provided no tools or frameworks in the former two categories are available. Consider, for example, studies with uncommon experimental designs and data resources that require custom data wrangling and visualization workflows. Such scenarios can be tackled by using either tabular data processing GUI's or custom-made scripts, alongside manual customization in visualization software such as Cytoscape.^163^ Most visualizations highlighted in this review fall into the first two categories, while custom uses of cutting-edge visualizations often fall into the last category, at least until made available as tools for easy reuse.

### 4.1. Raw data & feature processing

**Fig. 11:**
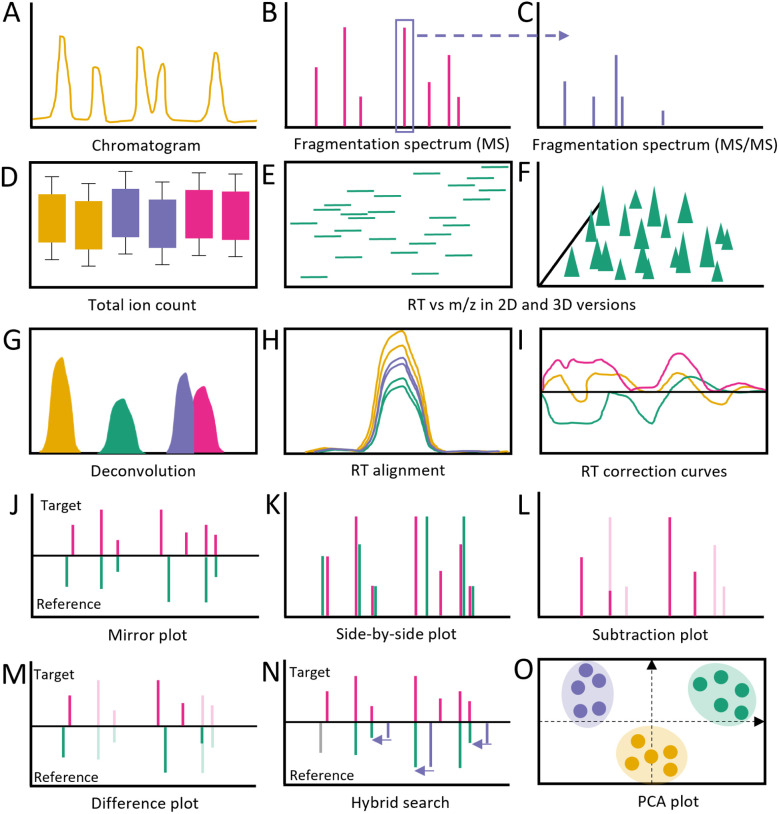
Commonly used visualization during raw data processing & spectral comparison. (A) Chromatogram. (B) Spectrum on MS level. (C) Fragmentation spectrum on MS/MS level. (D) Total Ion count for each sample, colors could be indicative of treatment. (E) Detected peaks for retention time and *m*/*z*. (F) Detected peaks for retention time and *m*/*z* including their intensity. (G) Deconvolution. (H) Alignment of one peak across different samples after retention time correction; color could be indicative of treatment. (I) Retention time correction amount. Usually plotted with retention time being on *x*-axis and the deviation after retention time correction on the *y*-axis. (J) Fragmentation spectrum of a target metabolite compared to a reference metabolite. (K) Fragmentation spectrum of a target metabolite next to a reference metabolite. (L) Fragmentation spectrum of a target metabolite with fragmentation of reference metabolite subtracted. (M) Fragmentation spectrum of a target metabolite compared to a reference metabolite with differences highlighted, while exact matches are faded. (N) Fragmentation spectrum of a target metabolite compared to a reference metabolite including hybrid search. (O) PCA plot with individual data points highlighted according to treatment. Abbreviation used in figure: *m*/*z* = mass to charge ratio, PCA = Principal component analysis. RT = retention time.

The first step in metabolomics data processing is raw data inspection (Fig. 11).^164^ Raw data is often saved in vendor-specific file formats and can only be visualized in the corresponding software. Only limited open-source software exists that can visualize vendor-specific formats, *e.g.*, OpenChrome,^165^ while most software requires raw data conversion to open formats such .mzML, and .netCDF (or .abf for MS-DIAL specifically^166^) which usually contain information on samples, acquisition settings, spectrum and chromatogram lists, *etc.*

**Fig. 12:**
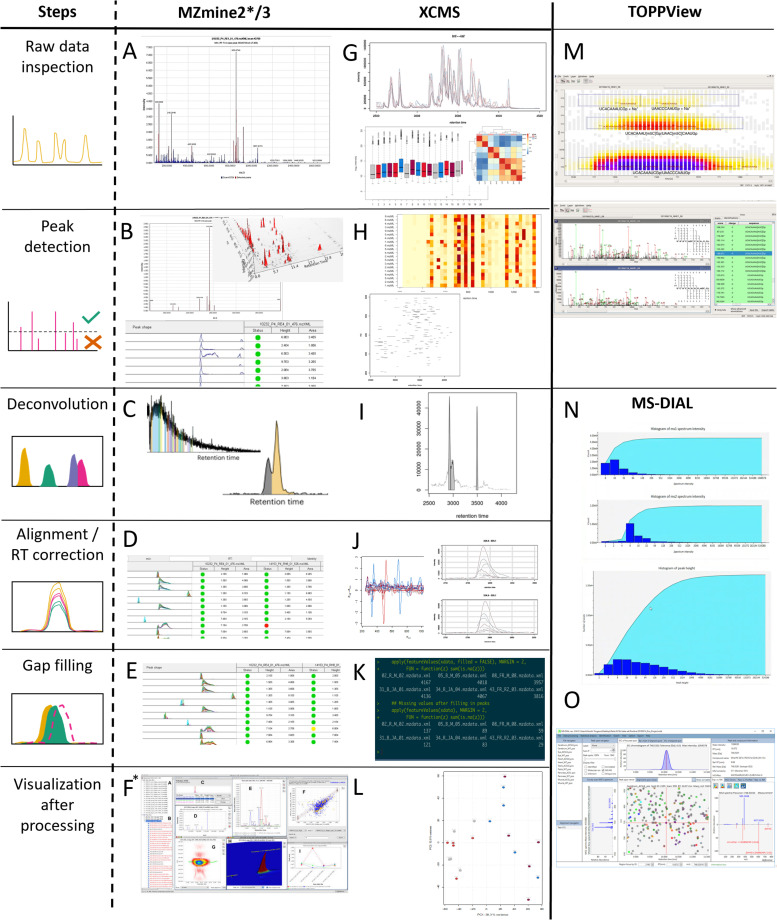
Example screenshots of selected software in metabolomics raw data processing for each step. The typical steps of metabolomics raw data processing start with the initial raw data inspection, followed by peak detection, deconvolution, alignments and retention time (RT) correction, gap filling, and final visualization after processing (left column). MZmine (A–F) and XCMS (R version; G–L) have visualization throughout all the steps, while TOPPView (M) and MS-DIAL have a raw data viewer (N). MS-DIAL exclusively has a final post-processing viewer (O). (A) MS fragmentation spectrum of raw data. (B) MS fragmentation spectrum after peak detection, 3D plot of RT *vs. m*/*z vs.* intensity, and peak table showing the peak shape for every feature. (C) Deconvolution as differently colored peaks in chromatogram. (D) Peak table after RT alignment showing in which samples the peak has been found (green dot) and in which ones not (red dot). (E) Peak table after gap filling. Red dots turn yellow if peaks are now included. (F) Final visualization after processing raw data. Individual windows show peak shapes, MS/MS fragmentation spectrum, chromatograms of multiple samples, scatter plot of peak areas of two samples, 2D and 3D plot of a peak (RT *vs. m*/*z vs.* intensity), and line graph of peak over multiple samples (*Screenshot taken from MZmine2 (ref. 176)). (G) Chromatograms of each sample and total ion counts, both colored based on treatment. Additionally shown correlation heatmap for the samples based on raw data only. (H) Heatmap with binned retention time on the *x*-axis and samples on the *y*-axis showing the number of detected peaks and a 2D scatter plot of identified peaks (retention time against mass-to-charge ratio per sample). (I) Deconvolution shown with line drawn between individual peaks in chromatogram. (J) Retention-time-correction line plot and chromatographic test-peak before and after alignment. (K) Table showing the amount of missing peaks before and after gap filling. (L) Bi-plot from principal component analysis. (M) Raw data viewer of TOPPView. Upper panel shows MS view (RT by *m*/*z*); each box is a peak with color giving indication of peak intensity, while the lower panel shows the MS/MS fragmentation spectrum. (N) Raw data viewer of MS-DIAL show bar graphs for RT, MS and MS/MS spectrum intensity. (O) Final visualization of MS-DIAL after processing including MS spectrum, Retracted ion chromatogram, Meta data, peakspot viewer, and mirror plot.

Raw data inspection can usually proceed from these export formats (Fig. 12). This step is essential for multiple reasons. First, it is possible to detect experimental errors or artifacts introduced during extraction and measurement. For example, a researcher may seek to locate expected peaks, such as internal standards or known compounds, within chromatographic overview representations to provide a quick and effective sanity check of experimental and computational processing results. Second, within the same chromatographic representation, the quality of samples can be inspected through assessing clear separation between peaks or co-elution of peaks. Third, initial insights into noise levels (baseline *vs.* peak height) and differences between batches can be gleaned by overlaying multiple chromatograms atop each other. Additionally, visual assessment of the raw data can guide the selection of parameters for raw data processing. For example, MS-DIAL portrays three histograms with MS and MS/MS spectrum intensity and peak height.^166^ Alternatively, XCMS (R version) provides additional sample-correlation heatmaps based on the chromatograms to allow users to quickly assess whether samples of specific treatments cluster together or not.^167^ XCMS further shows the total ion count across samples in boxplots. Moreover, there are software tools that provide visualizations of the raw MS/MS data, *i.e.*, TOPPView and GNPS dashboard.^168,169^ Such visualizations display the MS/MS fragmentation spectra with highlighted matching peaks to some reference.^170^. Additional efforts are also being made to ensure the quality of raw data in more objective ways.^171^ However, the current state of the art involves extensive and time-consuming manual evaluations steps assisted by overview visualizations.

The next step in the analysis workflow is to transform the complex raw data into (1) a feature table containing peak intensities or concentrations per sample for statistical analysis, and (2) a spectral data file such (usually a .mgf mascot generic format file containing at least the precursor mass, charge and *m*/*z* – intensity pairs) file for network analysis. Raw data processing in untargeted metabolomics is an intricate and potentially error-prone task.^162,172–174^ Large sample numbers, including blanks and pooled samples, need to be processed such that (a) large amounts of noise are removed and baseline corrected, (b) features are accurately detected (peak picking), (c) retention time and mass shifts are aligned across samples, (d) intensity batch effects are corrected for, (e) features are grouped to tackle adduct/insource and fragment/isotope redundancies, and (f) MS/MS fragmentation data are denoised.

The visualizations available during processing differ from software to software. For instance, XCMS (R version) and MZmine visualize processing results after each step, MetaboAnalyst has several plots the user can inspect upon choice, while software like MS-DIAL exclusively shows summary processing results at the end of processing.^158,166,167^ A recent Python-based package TidyMS also offers all steps from raw data leading to the metabolite feature table, with various visualization plots to assess the quality and impact of processing and filtering steps.^174^ To evaluate the success of denoising and baseline corrections, straightforward inspections of chromatograms can already be insightful.^175^ After such corrections, the baseline of the chromatogram should be flatter and noise peaks below the set threshold should be removed. Peak picking often happens at the same time as denoising and baseline correction. During this step, peaks across retention times and the *m*/*z* range are selected which can be represented in a 2D scatter plot of identified peaks where axes represent retention time and mass-to-charge ratio. This highlights retention time ranges in which many peaks can be found (should align with the chromatogram) and which mass-to-charge ratio ranges can be found at that given retention time (Fig. 11E). XCMS generates a heatmap with binned retention time on the *x*-axis and samples on the *y*-axis. Herein, color intensity shows the number of peaks detected. In MZmine, peak picking is displayed in a large table for each retention time and mass-to-charge ratio combination showing detected peak shapes. This allows for peak quality checking *via* peak shape inspection, *i.e.*, “clear peak” *vs.* hump, and corresponding manual curation *via* interactive editing. After peak picking, co-eluting peaks need to be separated by a step called peak deconvolution. In XCMS, this step is called peak refinement and is shown by drawing a line between the individual peaks in the chromatogram. For MZmine, the individual peaks are colored differently during this step.

Following deconvolution, peak alignment is performed to adjust for potential retention time shifts across samples. Again, the chromatogram can be used to visualize this step. Before alignment, peaks across samples show variability in retention times in the range of milliseconds to seconds, while after alignment, peak apexes for features found across samples should be at the same retention times. Often, a retention-time-correction line plot is generated to show by how much the retention time had to be shifted. Here, large deviations between original and adjusted retention times can be an indication of processing problems. XCMS allows for the selection of a few test peaks to visualize the changes before and after alignment. Alignment completion also leads to an update of the heatmap with the number of peaks. In addition, tabular data is updated with sample-specific columns. The columns contain green or red dot-based highlighting to show whether a peak was detected in that sample or not (Fig. 12 (MZmine alignment/RT correction)). Gap-filling is an optional step to infer peaks that were found in most, but not all samples of one group. Some peaks may be missed by peak detection as a result of low intensity and/or high background noise levels. This might be caused, among other things, by the natural variation of the sample, variation in sample collection, and extraction efficiency. XCMS provides only a table with the number of missing peaks before and after gap filling. MZmine shows the same table used during retention time alignment, with newly detected peaks highlighted in yellow.

After having completed the pre-processing of the data, a feature table is generated. Feature tables contain retention time, mass-to-charge ratio, and sample-specific peak intensity for each feature. However, each processing tool may add additional tool-specific information. The final step to check whether processing was completed correctly. To spot potential artifacts that may have been introduced by processing, the feature table is plotted using a Principal Component Analysis (PCA) plot (also known as biplot).^177^ PCA is an algorithm used to extract the primary axes of variability from high-dimensional data. Here, high-dimensional data is projected onto orthogonal axes such that each axis captures a maximum amount of variability. This projection allows high-dimensional data to be displayed using a two-dimensional scatter-plot using the first two of these axes, usually allowing insights into major trends of variability in the data. It is thus possible to see whether samples from one treatment are clustering together, what the intrinsic variation of one cluster could be, and whether treatments are different from each other.

To work with both MS feature tables and MS/MS spectral data in the same analysis, software suites like MZmine enable the selection, if available, of MS/MS spectra for each detected MS feature, based on user-defined criteria. For example, one can choose the MS/MS spectrum with the most intense base peak, or the MS/MS spectrum closest to the apex of the MS chromatographic peak. In TOPPView and other tools, chromatogram views with marked spots where MS/MS spectra were acquired are also available to spot-check for any obvious artifacts. These could include the repeated triggering of MS/MS acquisition for the same *m*/*z* value.

In this section, we have provided a birds-eye overview of the complex data processing steps in untargeted metabolomics, and how visualization plays a critical role in the manual validation of processing results. We underpin the important role of visualization to guide users in making informed choices and checking for obvious artifacts in both data acquisition and processing. The wealth of visualizations available to assist researchers in validating their data highlights how important data validation is to field.

### 4.2. Comparative spectral analysis

In untargeted metabolomics, LC-MS data alone are insufficient to achieve comprehensive structural insights for most measured compounds. Here, tandem-mass spectrometry (MS/MS) is used to generate fragmentation spectra revealing critical information for chemically meaningful spectral comparisons. Quantitative metrics to assess spectral similarity are a key to comparing large amounts of MS/MS spectra. Such spectral comparisons are used in a one-*vs.*-many situation to search against reference libraries or datasets, or in a many-*vs.*-many situation to discover relationships within datasets. In this section, we will focus on the role of visualization in validating library match information (one-*vs.*-many) and in data organization and exploration (many-*vs.*-many). In addition, we will discuss cutting-edge applications of visualizations making use of machine learning-based similarity scoring and whole reference library context visualization.

#### 4.2.1. One-*vs.*-many: library matching & spectral match validation: state of the art of visual tools

Assigning structural information to metabolite features is a key step in LC-MS/MS untargeted metabolomics workflows. The topic of metabolite annotation and identification in LC-MS/MS is reviewed extensively by others.^178^ We note that different metabolite identification levels exist that describe the level of confidence in the assigned structural information.^179^ Typically, library matching is done first, *i.e.*, the comparison of experimental spectra against in-house or public reference spectral libraries with the goal of either (i) identifying the query spectrum's chemical identity, or (ii) annotating the query spectrum by providing highly similar analogue structures based on mass spectral comparisons.^180^||||There are many different interpretations of the terms identification and annotation in the field of untargeted metabolomics (*e.g.*, ref. 179 and 181). To avoid any confusion, we define our particular differentiation between identification and annotation in this review. With identification, we refer to the process of determining the identity of the chemical species underlying the measurements of a feature. This is usually done using multiple lines of experimental evidence and qualified by degree of certainty, *e.g.*, levels of identification.^182^ With annotation, we refer to the much more general process of gaining any (putative) insights into otherwise unknown features. Insights can take the form of putative analogue matches, putative chemical class annotation, or putative substructure annotation. Analogue matches can serve distinct purposes; either as starting points of structural hypothesis generation, or as a means of prioritizing features of potential interest for experimental follow-up. In some cases, analogue matches can even be sufficient to answer research questions. For instance, when merely aiming to determine chemical compound classes. It is important to stress the duality of identification and analogue-based annotation, where identification serves the principally straightforward goal of determining compound identity, while annotation is best seen as a step within a prioritization workflow. That is, annotation allows gaining approximate insights into the chemistry of spectra, which in turn allows researchers to determine which compounds are promising for in-depth study. Annotation is thus more liberal and provides more comprehensive coverage, but also tends to be more vague and less reliable than identification, though even the latter requires an ontology of levels of certainty.^182^

**Fig. 13:**
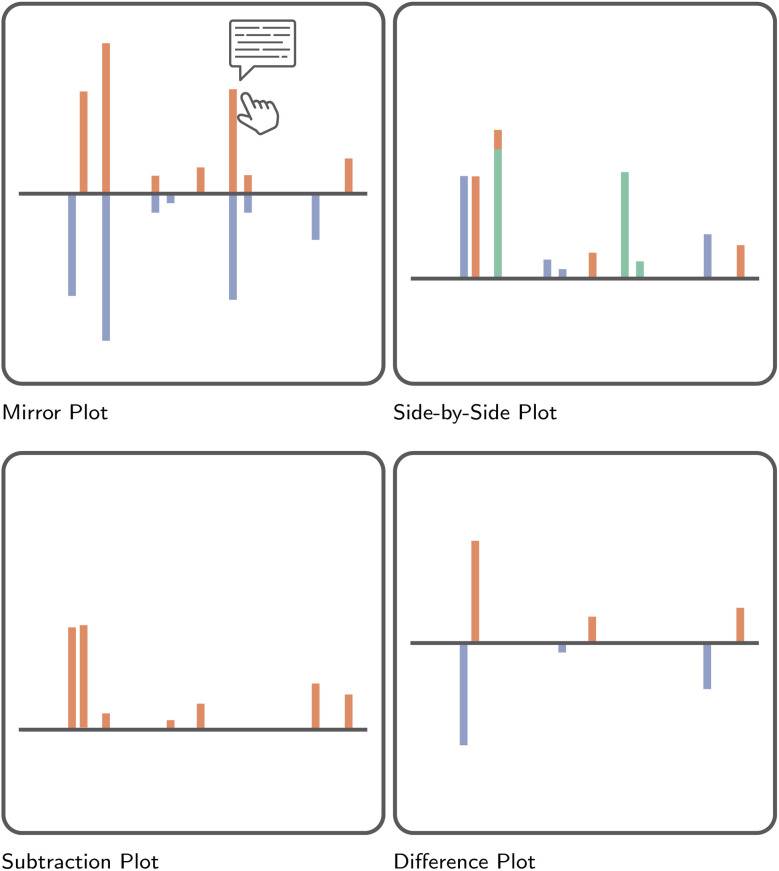
Spectral comparison plots usually portray two MS/MS fragmentation spectra *via* their mass-to-charge ratios and normalized intensities. The aims of pairwise spectral comparisons are (i) to assess library match quality, and (ii) to gain structural hypotheses within annotation propagation endeavors. From a visualization perspective, spectral comparison plots employ a wide variety of lower level “how's”. Specifically, they make use of (i) Encode → Arrange, displaying spectra by mass-to-charge ratio, (ii) Introduce → Annotate, where mass fragments may be automatically or manually annotated with chemical formulas, (iii) Facet → juxtapose/superimpose, where the two spectra are shown either side by side, in inverted *y*-axis, or contrasted to one another *via* subtractive approaches, (iv) Manipulate → Select, where additional fragment specific metadata such as precise mass to charge ratios or auto-generated chemical formulas may be viewed *via* hover tooltips, (v) Manipulate → Navigate, where zooming and panning allows for viewing areas of the spectra in more detail. The low level goals of the comparative plots are (i) Query → Compare, for the comparison of the spectra, (ii) Consume → Present, to present the mass spectral data, (iii) Produce → Annotate, where the user wants to gain insights into fragments and their likely chemical identity, and (iv) Search → Explore, where the user starts the spectral comparison with little knowledge of the fragmentation or characteristic fragment ions of both spectra. Spectral comparison plots thus provide a myriad of functions and encodings that assist data analysis well beyond spectral similarity scores.

In line with the importance of the identification and annotations tasks, a larger number of tools exist that streamline library matching for users, including among others GNPS(2), MS-DIAL, MZmine(2/3), and MS2Query.^156,158,166,180^ Focusing on library matching visualizations, we note that the current standard approach is one of tabular library hit representation, where either the best, or the top-*k* best hits are represented in a tabular ranked list. The two primary uses of visualization in annotation are the data integrative network visualizations discussed below, and visualizations serving match validation purposes. Within the library matching tools themselves, visualization appears in the form of spectral comparisons between query and reference spectra, as well as reference spectrum structure visualization *via* structural glyphs. It is important to stress that while library matching is a largely automated task, match validation often remains a manual expert task. Here, spectral matching to reference libraries as summarized by scores is a simplistic and insufficient summary for validation and thus often supplemented with a direct spectral comparison of query and reference spectra using visualizations such as spectrum and mirror plots (Fig. 13). When making use of public data repositories for matching, it is vital to have access to ample metadata for this validation. Relevant metadata may include the reference's analytical instrumentation and its settings (machine type and accuracy, mode, *etc.*), spectral origin metadata (sample type, processing, annotation type, *etc.*), and structural glyphs for the reference. Additionally it can be helpful to include chemotaxonomic data, like the species a metabolite was observed in before. This kind of information can be extracted from public databases like Lotus.^183^ Tools like plantMASST or microbeMASST^184,185^ take this one step further by performing library matching to unannotated spectra for which chemotaxonomic data is available. Incorporating this kind of data alongside spectral comparisons can help the user with judging if an annotation is likely to be correct.

Within GNPS and GNPS2, each spectral library match can be spectrally compared to the query spectrum *via* mirror plots (Fig. 13 (Mirror plot)). In addition, reference library spectrum information such as the chemical structure annotation and corresponding collaborative expert annotation rating are visualized in integrated views.^186,187^ The inclusion of metadata into library data views is a crucial component of library match validation, prompting also the development of tabular overview representations such as in Gauglitz *et al.*^188^ Extensive query to library visualization capabilities are also provided with the NIST Hybrid Similarity search tool, providing mirror plots, differences plots, and subtraction plots (Fig. 13).^189^ Hybrid search mirror plots extend the concept of mirror plots to include highlighted fragments that match up to a mass shift, showing fragmentation overlaps even for fragments with mass differences as a result of molecular additions or subtractions between the complete molecules. In addition, structural glyphs are superimposed into the view, allowing toggling between the top-*k* matches in the library to provide insights into potential shared substructures within the analogue set. Substructure analysis is further assisted *via* MS-Interpreter, which provides a means of selecting substructures in a structural glyph and highlighting corresponding mass fragments and associated chemical formulas. Similar capabilities of spectral comparison under mass shifts and highlighting of substructures in structural glyphs is provided in ModiFinder.^190^ Fragmentation overview maps (*i.e.*, FragMaps) highlighting spectral overlaps such as the ones used in specXplore could also be useful in the context of analogue library search match list visualization.^191^

Individual library hit validation is done with subtle differences in intent between finding of matches for full structure characterization and obtaining partial matches in case of analogue searches. These differences become more apparent in the context of machine learning tools such as MS2DeepScore that allow broader matching beyond direct fragmentation overlaps.^192^ Here, extensive confirmatory spectral comparison is less relevant, but may yet be of use for evaluating the reasonableness of match(es). For instance, finding characteristic fragment ions, fragmentation motifs, or shared substructures within the top-*k* analogue matches may each provide validatory insights even in cases of lower fragmentation overlaps. Substructure overlap may also prove useful in the complex case of matching across ionization modes as done by de Jonge *et al.*^193^ The feature frequency histograms and annotated MS/MS spectral plots implemented in ms2lda.org (http://ms2lda.org/) are two examples of visualizing machine learning-based patterns in the context of the experimental data.^194^ These plots support the inspection, substructure annotation and identification, and validation of MS2LDA-discovered unsupervised mass fragmention patterns.^195^ They render the subsequent analyses less tedious, as a user is now able to quickly select the most interesting and reliable patterns, for example by the shape of the histogram. We are not aware of any other implementations of such visual strategies or potential novel alternatives for this purpose. Machine learning predictions, whatever their reliability to the case at hand, have proven that they can provide useful pointers to chemistry of interest and perform better than classical (fragmental overlap-based) scores. However, they leave an open validation challenge currently only addressed by manual expert validations (in so far possible given spectral information) or additional experimentation.

#### 4.2.2. Many-*vs.*-many: create new insights from relationships between spectra

**Fig. 14:**
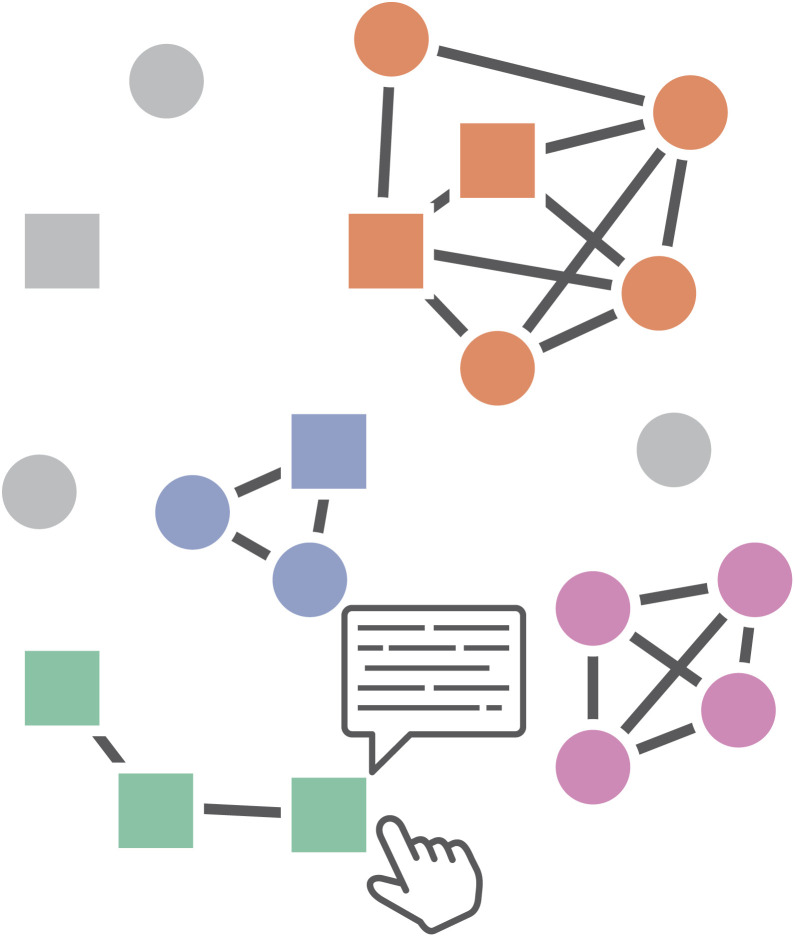
Mass spectral networks serve a wide variety of functions in the exploratory analysis of untargeted metabolomics data. The three main higher-level functions of molecular networking are (i) mass spectral data organization *via* the subdivision into molecular families, (ii) data exploration *via* interactive inspection of features and their interconnectivity, and (iii) as a data integration scaffold for analysis and presentation. Many different technical “how's” are used to make molecular networking as versatile as it is, including (i) Encode → Arrange, where MS/MS spectral features are arranged into node-link diagrams by cluster size, or in more recent developments overlaid onto a latent variable space, (ii) Reduce → Filter, where connectivity between features is limited *via* spectral similarity and topological constraints, (iii) Reduce → Embed, where mass spectral features are represented by node glyphs or more complex representations encoding additional information, (iv) Manipulate → Select, where hover information is used to provide node information, (v) Manipulate → Navigate, where panning and zooming is used to inspect broad areas or focus in on local areas, (vi) Introduce → Annotate, where additional spectral annotation information or library matches are introduced for nodes, and (vii) Introduce → Import, where statistical information, annotations, or experimental information can be included as color, size, glyph, or shade information. From a visualization perspective, the goals of molecular networking fall into the categories of (i) Produce → Annotate, where features and clusters of features are annotated, (ii) Produce → Derive, where node link diagrams are derived from spectral similarity data, (iii) Search → Explore, where nodes of interested are sought for inside node-link diagrams *via* their connectivity or annotations, (iv) Query → Identify, where once a node of interest is found, it's annotation and available information are sought for, and (v) Query → Compare, where spectral features are compared beyond the thresholded pairwise similarity-based edges. With such a variety of technical components and goals, it is no surprise that molecular networking is commonly used, used for different purposes, and modified into different workflows that facilitate particular analysis tasks in a more targeted fashion.

**Fig. 15:**
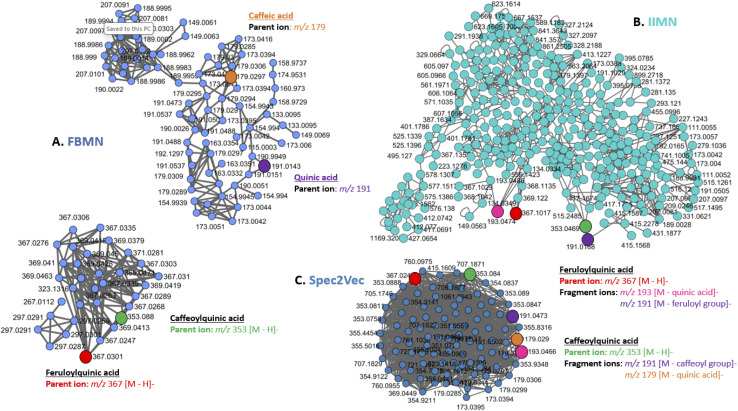
Example molecular networking families for ESI-negative MS/MS spectral datasets from maize leave samples (see also ESI†). Figures show sub-networks for (A) feature-based molecular networking (FBMN),^77^ (B) ion identity molecular networking (IIMN)^199^ and (C) Spec2Vec similarity-based networking^77,200^ generated from samples. The different figures reveal differences in the clustering of related ion species when different molecular networking approaches or similarity scoring methods are applied. The example data are from ongoing research on maize samples. Here, the highlighted compound structures are library hits for hydroxycinnamic acid (HCA) compounds of interest. Initial FBMN revealed high numbers of in-source fragmentation and separation of parent ions from the HCA compound class of interest which were expected to cluster together. Subsequent analysis using IIMN tackled the in-source fragment redundancy and the use of the Spec2Vec similarity score improved the clustering of the HCA features into one densely connected network. The complexity of the visual analytics process underlying the use of molecular networking is striking in this example, where different variants of molecular networking are iteratively applied to improve data organization within an overarching effort to make use of library matches to explore and annotate additional features.

The second application of spectral comparisons is one of spectral similarity-based exploratory data analysis. Here, spectral comparisons are used to organize heterogeneous metabolomics datasets into more homogeneous subsets and are usually visualized interactively for data exploration and presentation.^76,191^ The most notable family of tools in this context is mass spectral networking, generally referred to as molecular networking.^76,77^ Loosely put, mass spectral networking is based on an inversion of the observation that similar structures tend to fragment similarly, to similar fragmentation implying similar structures. While an oversimplification of fragmentation behavior, this approach has proven to be a powerful basis for exploratory data analysis in biological sample contexts where many compounds measured share biosynthetic pathways. At its core, the visual strategy of molecular networking involves three components: (1) the visual representation of spectral features as nodes, usually with embedded information, (2) the connection of these nodes *via* edges provided they exceed threshold similarity limits (*e.g.*, spectral analogue similarity thresholds) and abide by additional topological constraints, and (3) the usually implicit organization of nodes into disjoint subnetworks to be treated independently of one another. Molecular networking thus serves a double purpose both as a clustering tool and as an integrated data overview representation (Fig. 14).^196^ In most studies, molecular networking is used as an analysis and presentation tool with a complex and usually iterative visual analytics process (Fig. 15). It forms the basis of mass spectral data exploration, where it is used to identify clusters, or communities of features, often referred to as “mass spectral families” or “molecular families”. Relationships between the features in these families are used for structural hypothesis propagation through the network, so-called Network annotation propagation (manual or automated).^159,197,198^ In addition, the cluster network visualizations often serve as a flexible data integration canvas *via* the superimposition of additional information.

**Fig. 16:**
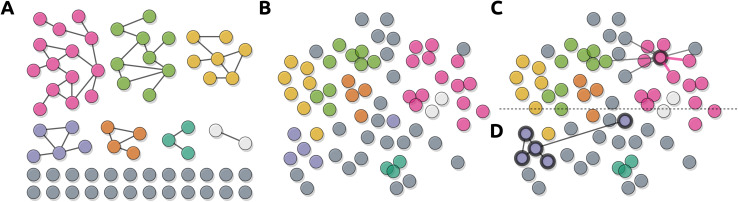
Representation of different means of representing mass spectral similarity data. (A) Similarity data are subjected to topological filtering and represented as (disconnected) node-link-diagrams (*e.g.* molecular networking^77^). (B) Similarity data are subjected to a two-dimensional embedding algorithm projecting them onto a two-dimensional plane, and represented using a scatter plot (*e.g.*, MetGem and specXplore^191,209^). (C) Interactive node-centric neighborhood displays using top-*k* edges for the selected node are added as an overlay to the embedding representation. This affords insights into connectivity beyond the embedding projection, and with adjustable top-*k* values, allows fluid exploration of intra- and inter-cluster connectivity (*e.g.* msFeaST^196^). (D) Edge overlays at specific threshold levels for clusters of features can pinpoint inter-cluster connectivity *via* the two-dimensional embedding projection (*e.g.*, specXplore^191^).

While network approaches offer one means of organizing and visualizing spectral data, they are not the only ones. In fact, as introduced in Section 3, the creation of 2D visualizations of a graph is full of complex challenges. Embedding-based approaches such as MDS (multidimensional scaling),^201^ t-SNE (t-distributed Stochastic Neighbor Embedding),^202^ UMAP (Uniform Manifold Approximation and Projection), PaCMAP (Pairwise Controlled Manifold Approximation),^203^ or tools to create minimal spanning trees such as TMAP (Tree MAP)^204^ can provide two-dimensional overview projections of mass spectral similarity as well. Such projections have the advantage that they tend to place “similar chemistry” close(r) to each other – in contrast to node-link diagrams where the placement of mass spectral groups is independent of their chemistry (Fig. 16).^191,196^ Choosing different tools and settings allows focus on more fine-tuned local grouping or global similarity patterns. Those techniques can thereby be tremendously useful in detecting feature groupings and trends in the data.^205,206^ Visual representation of embeddings makes use of scatter plots, where each feature is represented by a marker shape (usually points/filled circles) and placed into a (usually) two-dimensional grid. Embeddings are used to project neighborhoods in high-dimensional similarity space into neighborhoods in two-dimensional space. They represent a distortion of the high dimensional similarity space, however, in that no two-dimensional projection can accurately cover the full complexity of the high dimensional representation. Approaches such as MDS consistently perform better in distance preservation when compared to t-SNE or UMAP, yet fail to capture visually discernible trends in the data.^206^ Embeddings thus tend to work as a form of high-dimensional cluster mapping into two-dimensional space. This projection often also comes with its own interpretational complications, where for instance distance between point clouds in t-SNE can be meaningless.^207^ It should be noted, however, that network visualizations also constitute distortions of the similarity space in that the binarization of connectivity using edge thresholds or topological connectivity limitations employed in graph construction represents a simplification of the true pairwise similarity space. Despite this, both network and embedding representations are powerful tools for achieving overview insights into data.

Two-dimensional embeddings can be used on their own to serve as data overviews, but may also serve as node layouts for network representations. Here, nodes are positioned using the embedding coordinates rather than placed using traditional layout approaches that aim to avoid node overlaps or edge crossings (see Section 3). Depending on embedding settings or network settings, this approach can easily lead to visual problems in the form of node obstruction or cross-network edge connectivity. However, the approach does work well when using interactive edge overlays as done in specXplore or msFeaST (or visualization frameworks such as EdgeMaps), where edge overlays provide topological connectivity insights and path tracing capabilities beyond what is possible in a two-dimensional embedding alone.^191,196,208^ In addition to path tracing abilities, combining edge overlays can also highlight connectivity between clusters. Examples of tools enabling this are MetGem, where traditional molecular networking is connected to an embedding-based scatter representation with the intent of clarifying global trends or connections between molecular families,^209^ or specXplore and msFeaST where the network and cluster overlays are directly visualized onto the 2D embedding.^191,196^

#### 4.2.3. Mass spectral networking & embedding as a flexible data integration canvas

**Fig. 17:**
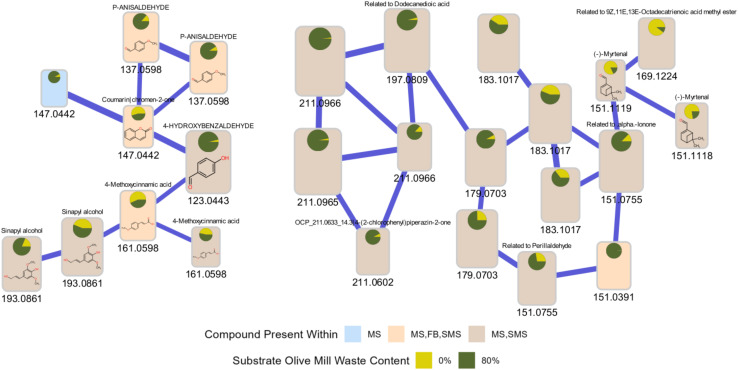
An example of a molecular networking infographic that uses pie charts to highlight differences in intensity of precursor ions of metabolites that are observed among sample groups. In addition to pie charts, network properties such as node color or size can be mapped to relevant variables to help differentiate the most relevant nodes. Putative identities of the features are provided with the name and structure of spectral library matches, *m*/*z* of the feature is shown below the node. Here, rectangular nodes represent individual metabolite features, the size of the nodes represents total feature abundance, and node colors indicate where the feature is detected (MS = mushroom substrate, FB = fruiting body, and SMS = spent mushroom substrate). The pie charts show proportions of feature abundance for samples with different concentrations of olive mill solid waste. Through Zenodo, an R script is available together with the example input data and expected output data that automates network processing and annotation, creating network visualizations such as the one shown here (https://zenodo.org/doi/10.5281/zenodo.12756070) Data was used from (ref. 219) – see also ESI.†

Networks and embeddings offer a powerful and customizable canvas for data visualization especially suited for pointing out relationships between features (Fig. 16). Networks excel at integrating different types of connectivity *via* edges and different types of features *via* nodes.^78,79^ Spectral similarity is a commonly used criterion for connecting nodes, but also specific mass differences, statistical association patterns (see Section 4.3), or shared fragmentation pattern motifs can be consulted to create connections.^210–212^ Networks allow for relatively easy integration of different types of features, a mixture of experimental and reference library spectra being one example.^191^ A multitude of node types and their topology can thus be evaluated from many different venture points. Besides their use as integrated exploratory data analysis tools, network and embedding representations find use as flexible canvases for data and hypothesis presentation (Fig. 16). Network connectivity data are often augmented with the manual super-imposition of sample context (*e.g.*, statistical treatment origins), feature context (*e.g.*, precursor mass-to-charge ratios, retention time, fragmentation spectra, *etc.*), and annotation information (*e.g.*, putative chemical class, structural glyphs for library matches) (Fig. 17). Various data are merged into one information-dense display for presentation. While network analyses appear to be used more frequently for this purpose, embedding-based scatterplots provide similar integrative flexibility on the feature level and occasionally find similar use.^213–215^

#### 4.2.4. How to get to integrated network visualizations?

The primary facilitators of spectral network creation are processing tools such as GNPS(2) or MZMine(2/3), among others.^77,158,163^ These tools provide node and edge lists and usually incorporate some amount of feature-specific data. While tools such as GNPS(2) or MZmine come with network visualization capabilities, those are often just the starting point for more in-depth analysis within more versatile network visualization tools such as Cytoscape. This way networks can be manually modified and styled, and styles may be exported for reuse.^163^ In addition to styling the visual appearance, additional script-based tools provide streamlined data integration for visualization within Cytoscape.^162^ Script-based workflows like MolNetEnhancer, also built into the GNPS platform, integrate molecular network data with annotations derived from other computational tools, such as *in silico* annotations from NAP and Dereplicator, and substructure annotations from MS2LDA.^159^ The MolNetEnhancer workflow uses these annotations to provide both a high level overview (*i.e.*, based on chemical classification) and detailed view (*i.e.*, based on substructure presence/absence) of the chemical diversity in molecular families visualized in the molecular networks. For developers working on custom dashboards Cytoscape.js and its Python or R interfaces *via* dash can be useful (Fig. 17). Tools like specXplore make use of these tools to join the outputs of pre-processing scripts to further augment and visualize network data in customized visualization and interaction workflows outside of the Cytoscape app.^191^ Here, network filtering settings are exposed in a custom dashboard alongside interconnected views offering visualization tool integration well beyond networks. The advantage of tools like specXplore is that they provide predefined and streamlined interactive workflows. This, however, comes at the cost of flexibility, where specXplore does not provide any of the customizability of the Cytoscape desktop app. When seeking a truly customizable workflow, a mix of manual Cytoscape modification and script-based approaches interfacing with the Cytoscape desktop app may prove useful. Script-based approaches make use of interface tools such as py4cytoscape to encode a pipeline for automatically and quickly creating network visualizations using customizable settings. These tools either make use of the Cytoscape REST API as done in py4cytscape (Python-based) or RCy3 (R-based).^216–218^ Their main advantage next to speed and reusability is transparency, where the script gives a clear outline of what processing and styling steps are applied to the data. In addition, by being connected to the Cytoscape desktop app, they allow for further manual customization of functionalities beyond the capabilities of the scripting tool used.

#### 4.2.5. The potential of reference library visualization

Network or embedding-based visualizations are often used to organize and explore experimental data (Fig. 16). Less common and arguably at the cutting edge of the field is the exploration of full mass spectral reference library data *via* spectral or structural information. Full reference library visualizations can be applied to gain an overview of the available data and how well it covers a chemical space of interest. In addition, it may be of interest to visualize matches for a query spectrum in the context of the reference library, that is, in what area of the chemical space covered by the reference library does the query spectrum find its matches, and what is their confidence? Library visualizations may also serve to make the scope of machine learning models more transparent by providing a view of chemical space coverage of the training data used. There are currently only a few tools dealing with library visualization. Here, it is interesting to note that while network representations currently dominate experimental data representations, library visualizations tend to make use of embedding scatter plots. For instance, in Kretschmer *et al.*^220^ structural libraries are represented using UMAP embeddings with chemical class overlays to showcase coverage aspects of chemical reference libraries. Chemical compound classes can be assigned to structures by automated tools such as ClassyFire^221^ and NPClassifier.^222^ Examples of dedicated tools for library data chemical space exploration such as WebMolCS,^223^ or ChemPlot^224^ take a similar route in that they merge (a) some form of two or three-dimensional library embedding, with (b) some form of scatter plot representation with structure class-based color overlays and/or hover overlays. The inclusion of click or hover-based information on library molecules *via* tabular information and structural glyphs is also routinely done in this context.^204,223,224^ Library visualization differs from experimental data visualization in the scope of the visualization task. For very large chemical spaces computational constraints need to be taken into account and tools such as TMAP provide alternative two-dimensional projections based on minimum spanning trees.^204^ We expect library representations to become increasingly important exploration and validation tools: as the reference library sizes increase, library hits become more numerous, and machine learning models will be used more and more to perform library matching. Hence, it will become increasingly important to discriminate between the quality of various similar hits, an activity that visualization can support.

### 4.3. Statistical analysis

Untargeted metabolomics data and its many unknown or unvalidated structural hypotheses has always presented researchers with a prioritization challenge; which of the hundreds to thousands of features should be prioritized for laborious in-depth analyses? There are two primary routes to go about this, (i) *via* spectral comparison and putative annotations reflective of a chemical species of interest, and (ii) *via* statistical prioritization of features with differential intensity patterns (sometimes referred to as differential ion current) across samples in carefully designed experiments. Differential intensity patterns are especially promising since they only require precursor MS information and can point to interesting chemistry irrespective of any chemical information about the features, provided there are suitable samples to compare over a relevant statistical contrast. Differential intensity patterns combined with putative annotations or mass spectral networking are especially powerful in finding suitable data subsets for in-depth inspection. In a similar vein, the use and inclusion of biological context data, such as bioactivity information, can provide additional prioritization refinements. In this section, we will first outline traditional approaches for prioritization using precursor intensities exclusively, followed by integration of the latter with mass spectral similarity information. Finally, we will discuss bioactivity measurement data which can be used and integrated to a similar effect. Rather than focusing on the details of the statistical methods and their assumptions, we will focus on use cases and visual representations for validation, analysis, and presentation.

#### 4.3.1. Statistical approaches for prioritization

The starting point of all statistical feature prioritization workflows are feature quantification tables with picked features that are aligned across samples. These can be obtained from pre-processing software such as MZmine discussed in Section 4.1. Batch effect correction from different LC-MS runs, but also from different sampling times or experimenters are usually applied as part of the statistics pipeline prior to any substantive statistical analyses. These corrections are needed for prioritization to be based on relevant biological signals and not on technical artifacts. A variety of different methods exists, some based on the generic structure of the data,^225,226^ others on internal standards,^227,228^ or quality control (QC) samples.^229,230^ To evaluate the successfulness of batch effect corrections, the data are frequently displayed in a before and after PCA plot with the data points colored by batch.^231^ For datasets that contain QC samples, a good indicator for batch correction success is the close and central clustering of QC samples with one another.^157^ In addition, no systematic batch trends should be visible in the PCA representation.

Once the data are batch-corrected, statistical analysis can proceed. Here, visualizations often serve as a basis for statistical outcome inspection and presentation. A first step could be to analyze the correlation between samples or between features.^232^ Frequently used correlation scores are Spearman, Pearson, and Kendall which produce sample associations usually visualized in a heatmap. Here, either sample-against-sample or feature-against-feature correlations are drawn to highlight similarities in intensity patterns. These similarity patterns in turn are used to study groupings in the samples and groupings in the features. Similarity groups in the samples can confirm consistent treatment variable effects while feature groups can indicate sets of features of potential interest for sub-selection and in-depth analysis. As common in the -omics fields, approaches for feature prioritization can be separated into univariate and multivariate approaches. Here, univariate approaches deal with each feature individually, either leaving out multivariate integration completely or delegating it to visual summary approaches. Multivariate approaches on the other hand, perform an analysis step that inherently considers a multitude of features at once, which is also reflected in subsequent visual representations. Here, we will briefly outline some common univariate and multivariate approaches and their visual representations.

#### 4.3.2. Univariate methods for prioritization

In the category of univariate methods, well-established scatter plots, bar graphs, box plots, and spline plots serve to visualize feature-specific information for selected features. Since untargeted metabolomics data are semi-quantitative unless suitable standardization approaches are applied, visualizations make use of relative quantities such as fractions of total intensity in the sample, or ratios of parts, for visualization purposes. The commonly used cumulative intensity pie-chart for features in different groups is an example of these strategies. Alternatively, data normalization approaches may be used in-so-far applicable ways to generate quantitative references, enabling a wider range of statistical and visual analysis methods.^233^ In terms of normalization, different methods are available which can be sample-specific (sample weight or volume), by total ion count, or by reference feature. Furthermore, it might be necessary to transform (log transformation) and/or scale (*e.g.* Pareto scaling) the data for compatibility with statistical models or visual representation purposes. The peak table before and after normalization can also be visualized by kernel density plots (line graph with intensities on the *y*-axis and counts on the *x*-axis).

**Fig. 18:**
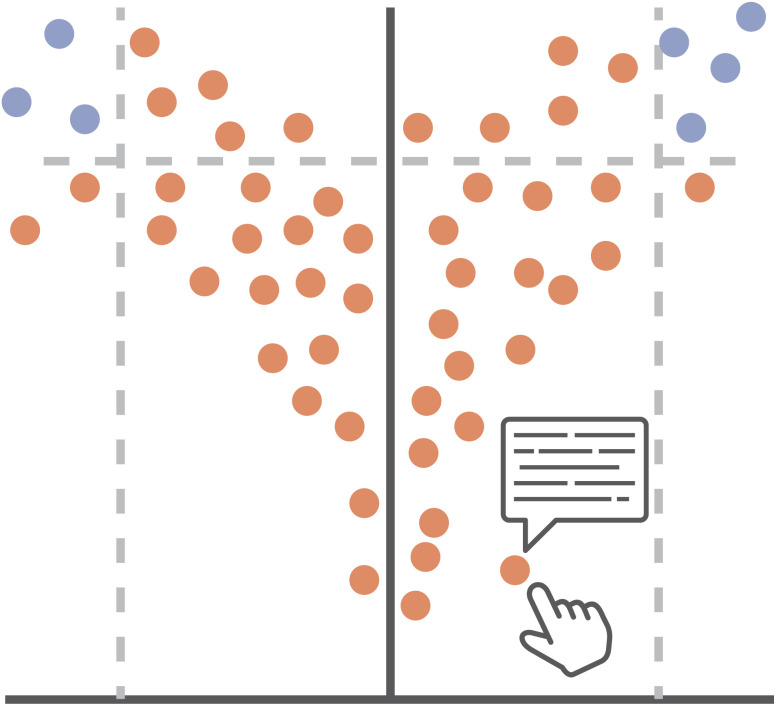
Volcano plots portray statistical summary data of repeated univariate tests on each feature, providing the user with a means of obtaining (a) an overview of the experimental impact on intensity profiles, and (b) a means of sub-selecting features with statistical differentiation across treatment groups. On a technical level, the lower level “how's” of the volcano plot are (i) Endode → Arrange, where *p*-values and fold changes are arranged by their magnitude, (ii) Encode → Map, where statistical significance and substantive effects are mapped to color/glyph/shade of points, and (iii) Reduce → Filter, in that features of statistical or substantive effect are emphasized over condensed areas of features of lesser effect. To a more limited extend, Manipulate → Navigate is used to allow inspection of crowded areas *via* panning and zooming. From a visualization perspective, the main lower level goals of volcano plots are (i) Query → Summarize, where intensity summary data are summarized using statistical measures, (ii) Consume → Present, where the plot provides an overview of the general trends across conditions, (iii) Search → Locate, where users can locate and identify features of interest *via* their position in the plot. Applying to a lesser extend is Search → Look-up, where users may want to assess known features of interest within the plot. Volcano plots are thus a prime example of Shneiderman's “Overview first, zoom and filter, then details-on-demand”. In applied analyses, they could be fruitfully combined with follow-up visualization or analyses in a myriad of ways, including the generation of promising subselections for in-depth visualization in heatmaps, or the highlighting of promising subselections in mass spectral similarity networks.

Spline graphs are flexible and smoothed-out (often model-based) line graphs that can allow for the in-depth inspection of intensity levels across time, or against a quantitative reference variable. This allows us to see overall patterns like steady increases over time, constantly changing abundances, *etc.* To compare single features over qualitative states, *e.g.*, treatment groups, box plots or violin plots can be used. In MetaboAnalyst, boxplots and violin-plots are always shown before and after normalization. Analysis of variance (ANOVA) is a parametric test to analyze if there is a significant difference in the means of a dependent variable between two or more groups. In ANOVA-like situations, each feature will have multiple difference parameters, indicating feature-specific trends for statistical contrast-specific differences. Depending on the visual strategy used, this may require a different representation for each contrast. In a MetaboAnalyst ANOVA plot, the individual peaks are plotted against the log-10 *p*-value.^160^ Repeated univariate testing results of fold changes across conditions can be summarized using so-called volcano and MA plots. For the volcano plots (Fig. 18), usually, log-2 fold-changes are plotted against log-10 *p*-values for each feature, providing an immediate overview of which features show high differences across the compared groups relative to their sampling variability. The MA plot similarly makes use of the log-2 fold change but this time it is plotted against the log-2 mean expression for each feature showing again the relative changes in metabolite intensities between two groups. In addition to these plots providing global views of effects across features, they are used to subselect features to highly promising candidates for in-depth analysis (Fig. 18). An additional representation of these data are heatmaps, which provide an overview of intensity patterns for numerous features across multiple or numerous samples. When combined with hierarchical clustering of the samples and/or features, heatmaps provide a view of potential intensity-based groupings in the features or samples. Heatmaps can be used to encode additional information and be ordered to portray an overview of the development of trends over complex experimental covariates such as time.^232^

#### 4.3.3. Multivariate methods for prioritization

In the category of multivariate analysis tools, PCA and PCoA can be used to create Biplots.^234^ When combined with sample information overlays in the form of color, these representations allow for overview insights. For instance, they depict straightforwardly whether the samples of different treatment groups separate in the primary axes of variability. Partial least squares (PLS) and PLS-Discriminant analysis (DA) work in a similar fashion, yet are optimized to accommodate particular sampling schemes such as regression (PLS)^235^ and two-group comparison (PLS-DA),^236^ where projections are optimized under consideration of the sample information. With PLS-DA, features that cause differences between two groups (control *vs.* test) are highlighted and can be selected for more detailed analysis and further visualization *i.e.* smaller heatmaps with only key features or single boxplots. PLS-DA might also be extended for multi-class problems. An extension of the univariate ANOVA is the combination with PCA for multivariate purposes. ANOVA simultaneous component analysis (ACSA) is used for the quantitative analysis of large multivariate datasets and allows for the explanation of variation caused by multiple factors or by time series.^237–239^ ASCA highlights major trends related to the factors and the interaction thereof. These trends are then visualized in trajectory plots (spline graphs) with the factors on the *x*-axis and scores on the *y*-axis for each of the factors and interactions. By plotting the leverage (*i.e.*, the importance of a feature's contribution to the ASCA model) *versus* the squared prediction error (*i.e.*, SPE, the goodness of the fit to the ASCA model) of each feature, one can study the model's quality and how the features contribute to it. Generally, features that are below a certain SPE and have high leverage follow the trends found in the trajectory plots. The final ASCA-related plot is a Venn diagram which shows the overlap of variables important for each of the factors and their interactions.

#### 4.3.4. Availability of statistical methods

Statistical analyses of untargeted metabolomics data require sample information metadata beyond the quantification table. Usually, manual tabular GUI or script-based pre-processing steps are required to ensure that the data are compatible with the statistical tool in question. Automatization of such alignment is only partially possible given the diverse nature of designs and means of documenting sample information. MetaboAnalyst provides semi-automated and convenient processing for 1-factor and multi-factor analysis.^157^ MetaboAnalyst can do filtering (depending on the number of features in the dataset), normalization, and then provide a large number of different statistical analyses. However, it requires at least three replicates per group and the setting selection for the plots is limited. MZmine and MS-DIAL, which are both raw-data processing software, can also provide the first steps for statistical analysis such as PCA and clustering.^158,166^ R packages such as ‘struct’ and ‘metabolomicsR’ provide complete freedom in the settings, but need more coding knowledge to perform the statistical analysis and visualization thereof.^240,241^

While statistical visualizations are often used to assist in data prioritization and sub-selection, they often come with their own limitations. Static heatmap representations of large datasets can quickly become unreadable or overwhelming. Here, sub-selections of the data, either using statistical measures or other measures such as spectral group information, can significantly improve visualization and thus the analysis process.^242^ Feature filtering cannot only improve statistical power, but also clarity of the visualizations used.^243^ Non-informative features suitable for filtering out can be divided into four categories: (1) low intensity which is close to baseline or detection level, (2) low repeatability across samples, (3) almost constant across groups, and (4) low feature quality when it comes to structure annotation, *e.g.*, lack of fragmentation making chemical annotation impossible. For each of these, filtering methods exist. Within interactive visualizations, such sub-selections can be automated and undone, affording high flexibility and convenience. In addition, selecting feature subsets for visualization through, *e.g.* volcano plots (Fig. 18), can improve subsequent detailed visualizations such as heatmaps. Thus, removing non-informative or irrelevant features enhances the overall visualization experience by focusing on the most relevant data.

#### 4.3.5. Integration of statistical information into mass spectral networking

Chemometric approaches provide prioritization means *via* statistical trends in precursor intensities across samples, while the previously discussed MS/MS comparative spectral analysis tools provide prioritization *via* chemical annotation and organization (see Section 4.2). Both approaches can be combined to form a powerful exploratory analysis framework. While the automatic integration of these toolsets is a topic of ongoing research and development, manual Cytoscape-based integration is already frequently used.^196,233^

To visually integrate chemometric information into spectral similarity networks, the style of nodes is often modified. For example, metrics from univariate analysis, such as log-2 fold-changes or *p*-values representing selected contrasts between sample groups, may be used to scale node sizes. The resulting network view simplifies the detection of differentially abundant molecular families, thereby aiding the discovery of chemistry that is relevant to the study system. Integration of chemometric information becomes more complicated when there is an interest in investigating multiple statistical contrasts. For this, a suitable approach may be a visualization strategy that allows for easy switching between views in which respective contrasts are used for node styling. Alternatively, simple charts may be presented on nodes to show how a feature's abundance compares between several conditions. Cytoscape offers various options for adding charts to nodes through the integrated “enhanced graphics app”,^244^ namely bar, box, heatmap, line, pie, and ring charts. The use of pie charts to include feature abundance information is illustrated in Fig. 17, which displays molecular families containing several features with relatively high abundance in samples with 80% olive mill waste content.^219^ A streamlined GUI for integrating common statistical methods with feature-based molecular networking data is provided in.^233^ Interpretation of molecular families that stand out in the spectral network due to the integrated chemometric information can be facilitated by including additional information layers, for example, chemical structure classes or bioactivity information (further discussed below) represented through node coloring.^78,79^ While networks are commonly used in the field to highlight statistical properties *via* node styling, they are not the only overview representation allowing for such encodings. Any visual encoding on the node level could be added to a scatter plot as well, and thus information overlays onto two-dimensional embeddings of feature similarity spaces can be used to highlight feature spaces with differential properties. An example of such an approach with interactive network elements is msFeaST, where a t-SNE embedding of pairwise similarity information is used with marker size encoding differential abundance of statistical abnormality properties.^196^

It is worth noting that not all network construction approaches in untargeted metabolomics are based on MS/MS spectral similarities. Statistical correlation networks, where edges represent precursor intensity correlations across samples, may also be useful for prioritizing feature groupings of interest.^78^ Such networks could in principle also be integrated with the mass spectral similarity networks discussed here in the form of multi-level networks with different edge types representing the type of connection between features.

While network visualization tools become ever more sophisticated and can integrate many forms of data already, they are not always the most suitable approach depending on visualization goals (see Section 2 and 3). Dedicated visualizations such as volcano plots can provide effective data overviews and sub-selection capabilities without the variability of network representations. They can also be coupled to network visualization workflows by serving as feature pre-selection filters, reducing the complexity of network visualizations. Longitudinal data may also be difficult to represent in networks, yet trivial to represent using spline graphs for selected features. A consideration of the goals and tasks for a given visualization can help simplify the visualization and provide clear visual underpinnings for decision-making and presentation 2.

#### 4.3.6. Beyond chemistry: integration into biological context

While prioritization is important to an exploratory field such as untargeted metabolomics, it is important to stress that the scientific discovery process does not stop at the prioritization once a set of features is selected or annotated. For example, researchers may be interested not just in which metabolites are enriched or correlated with their (bio)activity of interest, but also in which pathways they are part of and which metabolites may be responsible for a certain phenotypic variation. Such questions require the integration of additional information into analyses that originate from additional experimentation or data integration. Here, we will focus on pathway information integration and bioactivity data, while the following section deals in more detail with multi-omics integration.

We first consider the integration of bioactivity data with LC-MS/MS data. One of the simplest ways of achieving this is by using Cytoscape to manually layer information into molecular networks. For this purpose, metadata files are required for the molecular network workflows in GNPS. These can contain information regarding bioactivity and, if desired, additional data such as origin of the sample, taxonomy, growth conditions, among others. This way, all provided information can be accessed in Cytoscape and highlighted in different colors, shapes and sizes, in a fully customizable way that fits the user's needs and preferences in each situation.^245^

For a more automated approach, FERMO has been recently introduced as an interactive dashboard application that merges elements of raw visualization with the calculation of scores based on descriptive statistics.^246^ The dashboard allows prioritization of features based on putative novelty and/or association with bioactivity. The scores and other metadata of the samples and features are organized in tables on the dashboard and filters related to these parameters can be applied to help select molecular features of interest, for which the information can be exported, facilitating posterior analyses. Otherwise, they can be further explored directly in the dashboard, through integrated Cytoscape mass spectral similarity networks that reveal related features based on MS/MS similarity scores. Additionally, the distribution of each of these features across the dataset can be investigated through focused chromatogram views. We highlight that FERMO does not use the raw data to visualize the chromatograms; instead, it visualizes them as so-called “pseudo chromatograms”. In doing so, the tabular data becomes accessible to researchers in an appealing manner. Whilst this does not come without its limitations, such as distorted peak shapes for specific situations, it does allow for peak coloring based on the prioritization scores and metadata grouping specified, and it facilitates a light-weight summary file of the FERMO analysis that can easily be stored and shared. Furthermore, all information within FERMO is linked: clicking on an LC-MS peak in the chromatogram will highlight a spectral sub-network if MS/MS data are available, together with annotations if any were found.

Another tool that integrates bioactivity data with metabolomics profiles is NP Analyst.^247^ In this online platform, features are filtered according to scores that measure the strength and consistency of the predicted biological profiles. Then, the retained features can be visualized through connected views in networks, scatter plots, or community visualizations.^247^ The primary means of integration in NP Analyst is *via* node size and color encodings. Similarly, bioactivity-based molecular networking *via* GNPS allows for the streamlined mapping of bioactivity data onto their familiar molecular families.^248^ Another very recent framework that was just released is the NP^3^ MS workflow that encompasses many previous mentioned steps and data processing capabilities, including mass spectral library matching and bioactivity-based correlation of mass features.^249^ The NP^3^ MS workflow contains MS^1^ and MS^2^ (MS/MS) Viewers that aid users in setting processing parameters of the tool in the context of their data.

An alternative approach is used by MetaMapR, which includes different edge representations to highlight biochemical connectivity between features, thus moving beyond spectral similarity as the node connectivity property into more “pathway-like” connections.^250^ As such, it makes sense that in addition to bioactivity data integration, Pathway Enrichment analysis can help in interpreting metabolomic data and placing them into a biological context.^251^ Some methods, like gene set enrichment analysis (GSEA), over-representation analysis (ORA), and pathway level analysis of gene expression (PLAGE) have been successfully adapted from transcriptomics into targeted metabolomics.^252,253^ However, peak annotation is necessary for most of these types of analysis, constituting a limitation when working with untargeted metabolomics. One way of bypassing this is to perform additional annotation steps, *i.e.*, by using GNPS molecular family or MS2LDA substructure based grouping as a proxy for pathways as PALS does,^253^ or to use the mummichog algorithm, which was designed to not require preliminary identification of metabolites.^157,254^ More currently still experimental approaches include workflows such as msFeaST, which capitalizes on mass spectral similarities to form metabolite feature groupings, with implied structural and hence implied biosynthetic relationships, to provide feature-set level testing and prioritization to be integrated in network analysis.^196^

#### 4.3.7. Automatization *vs.* composability in statistical workflows

The effectiveness of visualizing statistics analyses in metabolomics depends on the computational implementation. Graphical User Interface (GUI) approaches for statistical analysis tend to be (a) strictly limited to specific frameworks, or (b) overwhelming in settings and can easily become opaque. Statistical analysis of any non-standardized fashion usually requires scripting in the form of data wrangling. Scripts for data wrangling have the huge benefit of allowing the sharing of processing steps and choices made transparently, as is sometimes done for GUI's built around command line interfaces, such as for MetaboAnalyst.^157^ In addition, they allow for the customization of processing steps to the case at hand. This is especially important considering the limitations of both statistical analysis tools and their visualizations. Visualizations can easily become undecipherable and overloaded when dealing with large datasets, often leading to the development of specialized tools for large data. In practice, researchers can gain a lot already from filtering their data towards promising sample and feature subsets, though arguably visual strategies are often providing the overviews and guidance required to do so. For example, representations like volcano plots can provide researchers insight into which quantitative cutoffs to use for subselecting data to be visualized in richer representations such as heatmaps of spline graphs. The endless possible combinations of filter strategies and study-specific requirements render these approaches best covered by script-based approaches. We emphasize that sharing such scripts with publications is an essential part of good scientific practice.

### 4.4. Multi-omics integration

Single omics applications such as metabolomics have been useful to elucidate molecular signatures underlying phenotypes of interest. However, using only single omics platforms, underlying mechanisms and patterns of molecular interactions for different phenotypes typically remain elusive. Integration of multiple omics layers enables to capture sequence of events leading to a specific process, state or phenotype.^255^ In this section, we provide an overview of how metabolomics data gets integrated within multi-omics studies, with a focus on the visualization approaches and challenges encountered in dealing with such complex applications that combine various omics data sources.

#### 4.4.1. Recent developments in multi-omics tool development

Within the last decade, the integration of untargeted metabolomics data with other omics datasets has been applied across various fields. For instance, paired omics analysis has been used to rank gene-metabolite links for natural product discovery.^256^ In complex systems where gene expression is regulated at multiple levels, such as eukaryotes, integration of transcriptomics with metabolomics in the form of gene co-expression networks provides an association-based strategy to detect co-expressed genes and co-abundant metabolites in a particular state.^257^ Metabolites interact closely with proteins in the cell, serving as substrates, co-factors, enzymatic products, allosteric regulators of enzymes, transmembrane receptors, or transcription factors. Thus, combining proteomics and metabolomics datasets offers a unique chance to gain new insights into the physiological processes regulated by genes on a system level.^258^ Lipidomics is regarded as an independent omics-based science due to the lipids' inherent structural diversity in cells, tissues and organisms. Integrating the metabolome and lipidome offers a comprehensive map of the metabolic landscape, enabling detailed network analysis to identify crucial metabolic drivers, for instance in disease pathology.^259,260^ Similarly, metagenomics studies can provide links between microbiome and metabolome data by associating microbial taxonomic groups with the surrounding metabolic variation.^261,262^ Taxonomic information can also expand metabolic network annotations.^263^ Importantly, single-cell multi-omics data has been widely employed to identify reliable associations between omics data by identifying cell type-specific signal.^264^ Spatially resolved transcriptomics in combination with MS imaging have allowed spatial multi-omics analysis.^265,266^

#### 4.4.2. Network-based multi-omics data visualization

Network-based visualization of multi-omics data is a common method used to link features from various omics sources. Individual features, such as genes, metabolites, or proteins, are depicted as nodes connected by edges based on mutual relationships. Network creation and analysis are key areas focusing on constructing high-quality networks and interpretation. Effectively navigating through these networks is essential for interpreting multi-omics data, especially since networks derived from large, heterogeneous datasets can be extensive. Unsupervised integration methods can be used to build associations between omics features, such as the correlation-based 3Omics^132^ or Similarity Network Fusion (SNF).^267^ Common challenges faced by these methods include the interpretability and computational scalability for larger networks. One solution is to reduce the number of input features from individual omics using tools based on unsupervised (MOFA)^268^ or supervised (DIABLO^269^ and mixOmics^270^) models, which effectively remove non-significant associations between features. This reduced feature set can then be used to build a highly refined multi-omics network. Additionally, deciphering significant relationships and generating hypotheses from a complex hairball of nodes and edges can be challenging. To address these challenges, various graph clustering algorithms have been developed including Markov Cluster Algorithm^271^ and ClusterONE.^272^ These algorithms capture similar interaction patterns between nodes and enable fast visualization of more meaningful subnetworks. Due to the high dimensionality of large multi-omics data, many data integration methods depend on dimension reduction techniques. These techniques look for dimensions along which the variance of the data is maximized. Pierre-Jean *et al.* (2020), have reviewed dimensionality reduction and clustering methods for multi-omics integration in great detail.^273^

The aim of visualizing the results obtained by unsupervised and supervised models is to prioritize and locate features by highlighting the features that contribute to most of the variation between treatments/groups/phenotype. With the rise of machine learning methods as the new state-of-the-art approach, several studies have explored these methods towards multi-omics integration.^274^ Specifically for network-based multi-omics analysis, graph-based machine learning tools offer a reliable methodology for data integration and for improved visualization.^275^ For instance, Alghamadi *et al.* developed a method based on a graph neural network model to infer cell-wise fluxome by combining single-cell RNA sequencing data with metabolomics.^276^ Deep learning methods excel in modeling and predicting outcomes from large-scale data where explicit relationships between data points may not be known or are too complex. However, they present interpretation challenges. Additionally, deep learning methods do not inherently handle the integration of structured domain knowledge or explicit modeling of relationships between entities, which are vital in multi-omics analysis.^255^

#### 4.4.3. Knowledge graph-based data integration

**Fig. 19:**
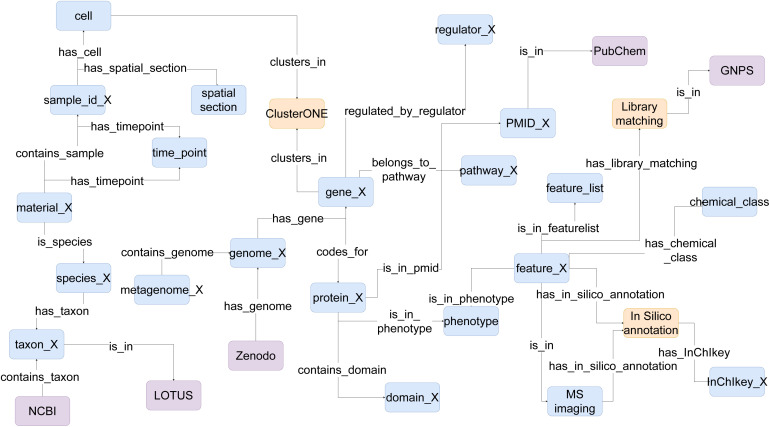
Example of a knowledge graph network integrating multiple omics data layers. Nodes are categorized and color-coded as follows: blue nodes represent ontology terms related to experimental design (*e.g.*, sample_id_X, time_point), phenotype, genomics (*e.g.*, genome_X, gene_X), and metabolomics (*e.g.*, feature_list, chemical_class). Orange nodes denote ontology terms for methods and analytical tools (*e.g.*, Library matching, ClusterONE). Purple nodes correspond to databases and other data sources (*e.g.*, Zenodo, NCBI). Edges between nodes represent relationships *via* ontology terms, facilitating the extraction and standardization of information across the graph.

In the context of multi-omics data integration, the ability of knowledge graphs to link heterogeneous datasets transparently and interpretably is especially valuable.^277^ Knowledge graphs provide another basis to analyze multi-omics data by leveraging and integrating information from ontologies of heterogeneous data. Knowledge graphs allow to store, extract and perform inference across multi-omics data while providing flexibility in integrating and normalizing the omics layers, thanks to a sample-centric approach and semantic enrichment.^263^ In a multi-omics knowledge graph, instances from different omics datasets as well as biological annotations such as taxonomic^278^ or pathway^279^ information can be used to extract relevant system-wide biological signals and their inter-dependencies as visualized in Fig. 19. Knowledge graphs can also be refined thanks to the use of publicly available multi-omics knowledge from public multi-omics datasets.

Multi-omics can help refining and extending metabolic networks by finding biologically relevant connections based on various omics data. Different multi-omics types have the potential to represent the complexity of different biological systems, *i.e.*, of prokaryotes and eukaryotes. Early and late-stage integration of omics data provides different strategies to navigate complex relationships among datasets. Knowledge graphs provide flexibility as they allow researchers to navigate all layers simultaneously or separately. Choices on how to integrate multi-modal datasets can influence and facilitate visualization of metabolomics data with biological annotations. Furthermore, visualization of multiple omics simultaneously can inform what integration strategy and analysis method to adopt for extracting relevant signals.^280,281^

## 5. Developer roadmap

Data integration, processing, analysis, and corresponding visualization challenges are ubiquitous in the field of untargeted metabolomics. Vendor specific and open source software are vital to the field, and without them, untargeted metabolomics would not exist. However, it is difficult to get funding for software development, let alone maintenance. Senior developers and visualization experts are rare, with open source development being largely left to MSc students, PhD candidates, and junior researchers, many of whom start their development work without any background in software development or data visualization. At the same time, researchers in the untargeted metabolomics and chemistry fields can be quite demanding, wanting cross-platform capabilities or web-hosted tools, as well as full graphical user interfaces allowing users with no scripting knowledge to access tools easily. In this section, we discuss the difficulties of open-source software development and give recommendations we believe could improve the development and accessibility of visualization tools.

### 5.1. Collaborate with experienced developers and visualization experts

The untargeted metabolomics field would profit from less solo development and more collaboration to include not just domain experts, but also data visualization experts and experienced research software engineers. The inclusion of data visualization experts could help developers make use of user-centered design processes and thus define more explicitly and accurately the “What? Why? How?” of the visualizations included in their workflows. Such an approach could allow for expanding the general utility and reliability of the workflow by making visualization capabilities a primary consideration rather than being treated as “pretty pictures”. Senior software engineers can help development-workflow robustness, and guide development into more robust and maintainable directions. Likewise, domain experts should be included in the development and testing of the toolsets produced *via* conference workshops based on prototypes.

### 5.2. Establish core workflow utility tools and common data structures for developers to use

The development of clearly defined core data structures for each tool to support would greatly assist the development cycle. Tools like matchms^282^ and MZmine3 (ref. 158) can provide a number of core and common data formats and conversion methods. The existence of these kind of tools works like cable adaptors, allowing for the translation of the output of one tool to serve as the input for another. However, a great number of formats, including formats that sometimes include certain information and sometimes do not, can still complicate analyses. It would be beneficial for the field to move towards human-readable, easily parsable, and standardized formats with clear entry expectations and formatting. For example, csv tables and json formatted data are easy to read, readily supported by almost any programming language, and have high-quality importing and exporting functionalities available. When combined with agreed-upon standard schemas, these formats can make data and data intermediates easily available for a plethora of development workflows. In addition to formats, the use of unique and consistent feature identifiers can greatly assist with integrative information mapping to and from spectral data.

### 5.3. Provide shareable interactive visualizations

Scientific communication using infographics is a critical component of scientific discourse. With rising requirements for data sharing such as fair data principles, it only seems appropriate to allow sharing of interactive visualizations. Ideally, snapshot views used to make certain claims or which serve as the basis for infographics should be shareable to allow reviewers and readers to inspect the conclusions in the context of the data. Sharing of such visualizations with corresponding settings or Jupyter notebooks can further increase transparency.

### 5.4. Focus on developing customizable workflows & composable tool chains

The field of metabolomics is a rapidly developing field with new measurement platforms, workflows, and machine learning models published frequently. There is a need for both rapid and robust development of tools and corresponding visual capabilities. We anticipate that encouraging developers to develop more decoupled, specific tools, as well as to design customizable functionalities to inject into larger workflows such as the one of GNPS(2), MZmine, or MetaboAnalyst, would allow for rapid development of utilities to be ultimately included within larger workflow integration packages. This would allow developers and scripting-proficient users to make use of cutting-edge software, while non-scripting users could profit from robust standalone implementations being continuously integrated into larger workflows. The MetaboAnalyst GUI workflow is an interesting case study of this, where a GUI is provided around an R package infrastructure allowing users to export not just their settings, but also the scripts corresponding to their workflow. Such a script can be useful for reproducibility purposes, but also provide a means of customizing workflows by injecting new functionality into scripts or transferring the data intermediates into different visualization or analysis workflows. When script-based approaches are combined with hosted and containerized environments (*e.g.*, Nextflow as for GNPS2,^283^ or Code Ocean^284^), they can provide easy access and stable data science components for reuse by the community with less development and maintenance workloads.

## 6. Outlook

Since the first metabolomics experiments around the 2000s, visualization has been a vital component throughout the entire untargeted mass spectrometry-based metabolomics data processing and analysis workflow. Initially, data visualizations took place through opening raw format mass spectrometry files in vendor-specific software to perform tasks such as peak picking and quantification by selecting and measuring the LC-MS peak area. Such tasks placed visualization centrally by creating an interactive user interface in which the researcher made informative decisions on which parts of the LC-MS peak to include for quantification. For two decades or so, open-source alternatives have been developed for the community to perform an ever-increasing pallet of processing and analysis tasks. Interestingly, many aspects of the metabolomics analysis workflow have been “hidden away” from the user, oftentimes in an attempt to reduce the complexity of data analysis. Whilst this is a good motivation, this can hamper informed decision-making. For example, how can a user know that default settings are applicable to their use-case when the settings are abstracted away? How can a user check if the default settings of a workflow worked as intended for the studied data without visual insights? Steps that can be done reliably, consistently, and are robust to minor setting deviations can safely be “hidden from direct view” to make tools more user-friendly. However, when this is not the case, humans should be in the loop to make informative decisions. Here, we argue that visualization plays a major role in the critical quality control and parameter setting assessment steps.

Much like other elements in the computational metabolomics pipeline, metabolomics-related visualizations as described in our review is challenged by rapid developments in the field, including the increasing number of new analytical measurement platforms, increasing dataset sizes both in number of features and samples, increasing size and complexity of reference databases, and the increasing need for multi-omics integration. Despite their ubiquitous presence and use in the field, the development of visualizations largely remains a secondary consideration within the larger workflow development process. With this review, we aim to highlight not only the importance and ubiquitous use of visualization, but also the wealth of development opportunities and cross-pollination potential between the metabolomics and the information visualization communities. Given its importance, we argue that visualization should receive more dedicated design and development attention. While aesthetic considerations, domain-specific conventions, and good design principles certainly add to the appeal of visualizations, we stress that effective visualization is a measurable science, and can be put to the test. While user studies are unlikely to become common-practice in the field, they should be used more often to study and validate the performance of visualization and analysis workflows.^285,286^ Their inclusion could give focus to development processes and provide scientific underpinning to developments.

Since visualization is fundamentally tied to computational metabolomics data science infrastructures, our recommendations to improve visual tool sets and their accessibility are primarily addressed to developers. Dedicated visualization programs and their ultimate inclusion into widely used processing workflows provide an essential stepping stone toward widespread adoption of new and improved visual tool sets. We note that the evolving cutting-edge and widely customizable tools will likely remain exclusively accessible to researchers with scripting knowledge or computational metabolomics colleagues. To allow researchers to make use of these tools and incorporate them into their workflows, more tailored workshops on these tools and the use of scripting in general are needed. Nevertheless, we also stress that there is a responsibility for users to put effort in reading available manuals and documentation to broaden their understanding of settings and expected outcome.

As metabolomics continues to generate large volumes of data, visualization facilitates the identification of patterns and trends that might otherwise remain obscured. For example, metabolomics-based repository-wide searches and analyses are increasingly supported by tools like MASST^287^ that have resulted in dedicated well-curated spin-offs for microbes^185^ and plants.^184^ Such novel tools create the need for novel innovative ways of visualizing metabolomics data to put the search results into context. Here, the metadata curation facilitates the visual representation of the presence/absence of mass spectra across microbe and plant taxonomy. Overall, the development of open-source platforms and software, such as MetaboAnalyst, MZmine, GNPS and its dashboard, as well as matchms, has democratized access to advanced analysis and visualization tools, promoting broader adoption and innovation within the scientific community. These tools are designed to handle the increasing complexity and size of metabolomics datasets, providing customizable and interactive visualizations that cater to diverse analytical needs. Here, we also observe an increased amount of stapled workflows connecting tools for different stages of the metabolomics workflow. We highlight that generic tools like matchms or the myriad of MZmine export options will provide vital cornerstones for connecting different workflow components and visualization together. They can also form one of the building blocks of multi-omics analyses where effective visualizations will become increasingly important to prioritize the relevant data sections for further exploration and subsequent validation. Given the increased complexity and multilayer dependencies of many existing omics tools, a main threat to usability is the breakdown in compatibility of one essential package in a tool resulting in a complete crash. We argue that there is an increased need for funding agencies to specifically acknowledge computational contributions and fund those as well, *e.g.*, to not only develop new analysis tools but to also (be able to) maintain them.^288^

Integration of metabolomics with other omics sciences, such as genomics, proteomics, and transcriptomics, yields highly complex and multidimensional datasets. Interactive and user-friendly visualization-guided platforms will become increasingly essential to help researchers to explore their data seamlessly across omics layers and to identify relevant correlations and interactions across different biological layers. Future trends in multi-omics visualization should include the development of tools that can assess the effectiveness of novel algorithms in selecting relevant cross-omics connections. Here, machine learning and artificial intelligence will likely play an increasing role in providing more precise and dynamic visualizations. Advancements in virtual and augmented reality technologies may also play a role, offering immersive visualization experiences that can provide deeper insights into multi-omics data. As these trends unfold, the ability to visualize complex data interrogatively will be indispensable to drive discoveries and innovations in metabolomics and natural product research.

Altogether, we think that visualization will remain crucial in the metabolomics and natural products fields, as these fields will increasingly rely on processing and analyzing large amounts of heterogeneous data to create effective endpoints that serve as a starting point for further studies. During these processing and analysis steps, there are several points that require informed decisions using the “overview first, zoom and filter, then details-on- demand” principle to enable prioritization of relevant data sections that are linked to the research questions at hand. And sometimes, one image just simply speaks more than a thousand numbers!

## 8. Author contributions

K. Mildau: conceptualization, project administration, visualization, writing original draft, writing – review & editing. H. Ehlers: conceptualization, visualization, writing original draft, writing – review & editing. M. Meisenburg: visualization, writing original draft, writing – review & editing. E. Del Pup: visualization, writing original draft, writing – review & editing. L. R. Torres Ortega: visualization, writing original draft, writing – review & editing. K. Saurabh Singh: writing original draft, writing – review & editing. R. A. Koetsier: visualization, software, writing original draft, writing – review & editing. N. F. de Jonge: writing original draft, writing – review & editing. D. Ferreira: writing original draft, writing – review & editing. K. Othibeng: visualization, resources, writing original draft, writing – review & editing. F. Tugizimana: resources, writing original draft, writing – review & editing. F. Huber: writing original draft, writing – review & editing. J. J. J. van der Hooft: conceptualization, funding acquisition, supervision, project administration, writing original draft, writing – review & editing.

## 9. Conflicts of interest

J. J. J. van der Hooft is currently a member of the Scientific Advisory Board of NAICONS Srl., Milano, Italy, and is consulting for Corteva Agriscience, Indianapolis, IN, USA. All other authors declare no conflict of interest.

## Supplementary Material

NP-042-D4NP00039K-s001

NP-042-D4NP00039K-s002

## Data Availability

No data or code was specifically acquired or created for this study. The data used is either available in the ESI, or through the original publications. The code used to generate Fig. 17 is available from Zenodo: https://zenodo.org/records/12756071, together with the example input data and expected output.
