# Supplementary material for: Effective data visualization strategies in untargeted metabolomics
Source: Nat Prod Rep. 2024 Dec 2;42(6):982–1019. doi: 10.1039/d4np00039k (PMC11610048; doi:10.1039/d4np00039k)
Supplement: NP-042-D4NP00039K-s001 [file NP-042-D4NP00039K-s001.pdf]

Supplementary Information (SI) for Natural Product Reports.
This journal is © The Royal Society of Chemistry 2024

Supplement: Effective Data Visualization Strategies in Untargeted Metabolomics

Kevin Mildau,[a,‡,*] Henry Ehlers,[b,‡,*] Mara Meisenburg,[c] Elena Del Pup,[a] Robert A. Koetsier,[a] Laura Rosina Torres Ortega,[a] Niek F. de Jonge,[a] Kumar Saurabh Singh,[a,d,e] Dora Ferreira,[f] Kgalaletso Othibeng,[g] Fidele Tugizimana,[g] Florian Huber,[h] and Justin J.J. van der Hooft.[a,g,*]

July 30, 2024

1 Datasaurus & Anscombe data examples

	Dataset	Average of X	Average of Y	Standard Deviation of X	Standard Deviation of Y	Correlation between X and Y	Linear Model Intercept	Linear Model Slope	Linear Model R-Squared
1	Away	54.2661	47.8347	16.7698	26.9397	-0.0641	53.4251	-0.103	0.0041
2	Bullseye	54.2687	47.8308	16.7692	26.9357	-0.0686	53.8095	-0.1102	0.0047
3	Circle	54.2673	47.8377	16.76	26.93	-0.0683	53.797	-0.1098	0.0047
4	Dino	54.2633	47.8323	16.7651	26.9354	-0.0645	53.453	-0.1036	0.0042
5	Dots	54.2603	47.8398	16.7677	26.9302	-0.0603	53.0983	-0.0969	0.0036
6	High Lines	54.2688	47.8355	16.7667	26.94	-0.0685	53.8088	-0.1101	0.0047
7	Horizontal Lines	54.2614	47.8303	16.7659	26.9399	-0.0617	53.2111	-0.0992	0.0038
8	Slant-Down	54.2678	47.8359	16.7668	26.9361	-0.069	53.8497	-0.1108	0.0048
9	Slant-Up	54.2659	47.8315	16.7689	26.9386	-0.0686	53.8126	-0.1102	0.0047
10	Star	54.2673	47.8395	16.769	26.9303	-0.063	53.3267	-0.1011	0.004
11	Vertical Lines	54.2699	47.837	16.77	26.9377	-0.0694	53.8908	-0.1116	0.0048
12	Wide Lines	54.2669	47.8316	16.77	26.9379	-0.0666	53.6349	-0.1069	0.0044
13	X-Shape	54.2602	47.8397	16.77	26.93	-0.0656	53.5542	-0.1053	0.0043

Table 1: Datasaurus data summary statistics for all twelve datasets and the **Dino**saur data. Averages, standard deviations, correlations between X and Y variables, and linear model outcomes for a model of Y given X are close to identical for all datasets.

	dataset	Average of X	Average of Y	Standard Deviation of X	Standard Deviation of Y	Correlation between X and Y	Linear Model Intercept	Linear Model Slope	Linear Model R-Squared
1	Anscombe Dataset 1	9	7.5009	3.3166	2.0316	0.8164	3.0001	0.5001	0.6665
2	Anscombe Dataset 2	9	7.5009	3.3166	2.0317	0.8162	3.0009	0.5	0.6662
3	Anscombe Dataset 3	9	7.5	3.3166	2.0304	0.8163	3.0025	0.4997	0.6663
4	Anscombe Dataset 4	9	7.5009	3.3166	2.0306	0.8165	3.0017	0.4999	0.6667

Table 2: Identical anscombe summary statistics for four datasets, each with very different scatter plots.

0[a] *Bioinformatics Group, Wageningen University & Research, Wageningen, the Netherlands*
0[b] *Visualization Group, Institute of Visual Computing and Human-Centered Technology, TU Wien, Vienna, Austria*
0[c] *Adaptation Physiology Group, Wageningen University & Research, Wageningen, the Netherlands*
0[d] Maastricht University Faculty of Science and Engineering, Plant Functional Genomics Maastricht, Limburg, the Netherlands
0[e] *Faculty of Environment, Science and Economy, University of Exeter, Penryl Cornwall, United Kingdom*
0[f] *NAICONS Srl, Milan, Italy*
0[g] *Department of Biochemistry, University of Johannesburg, Johannesburg, South Africa*
0[h] *Centre for Digitalisation and Digitality, Düsseldorf University of Applied Sciences, Düsseldorf, Germany*
0‡ These authors contributed equally to this work (shared first authorship).
0* Corresponding authors: kevin.mildau@wur.nl, henry.ehlers@tuwien.ac.at, justin.vanderhooft@wur.nl.

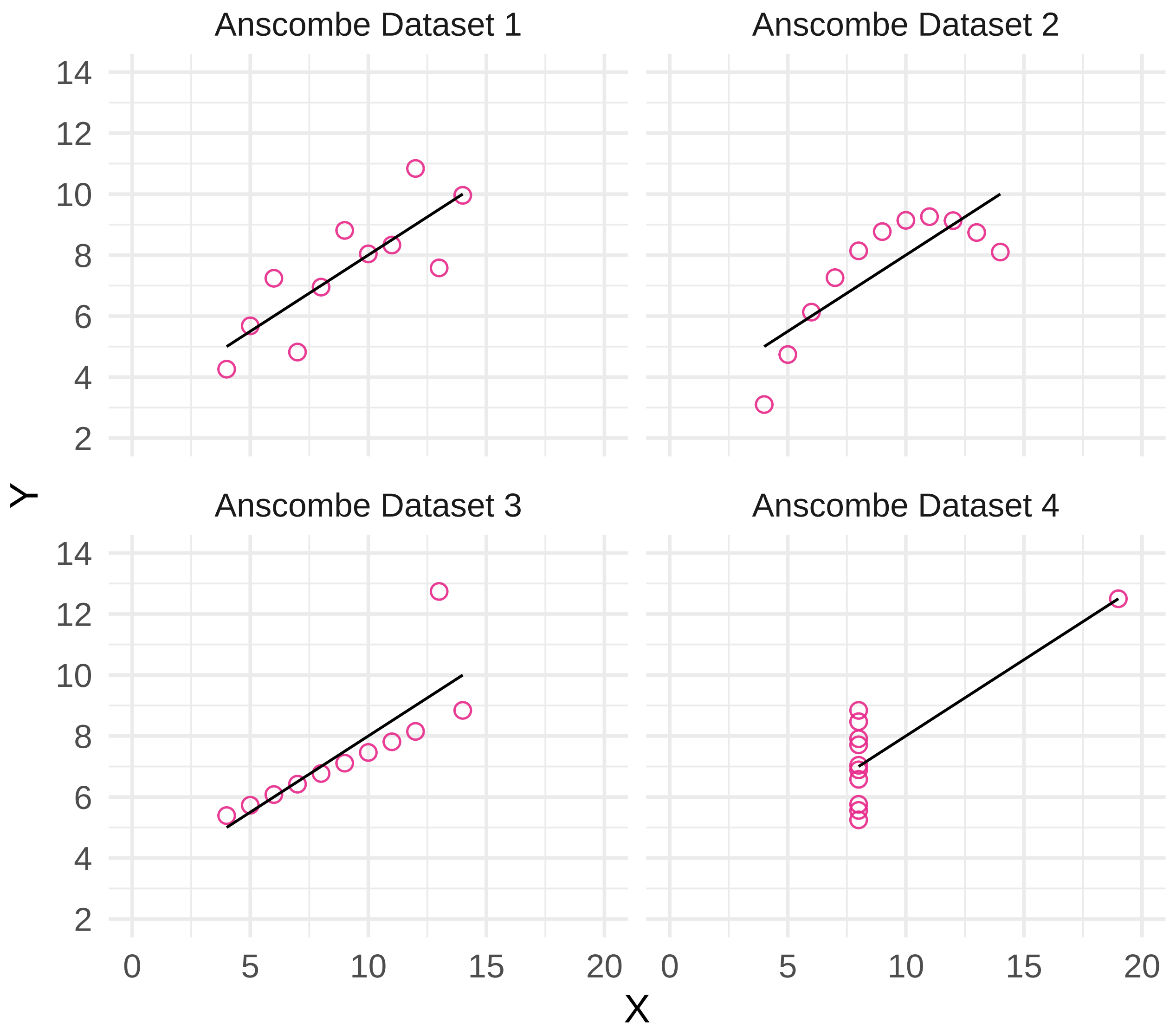

Figure 1: Anscombe data scatter plots. The four datasets show obvious differences in this representation that cannot be gleaned from summary statistics, and are very difficult to read from tabular data.

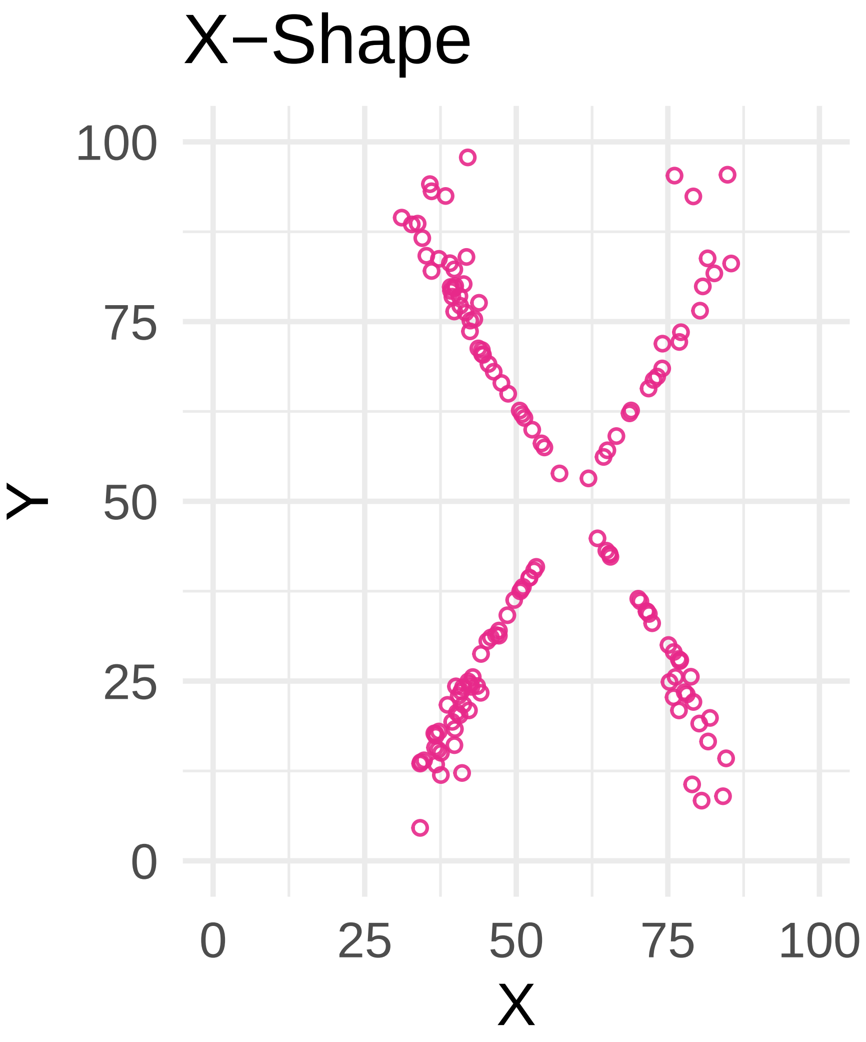

Figure 2: The X-shape dataset from the datasaurus example. As for the other datasaurus datasets, this is indistinguishable from the **Dino**saurus or any of the other datasest via common summary statistics (see table 1)

2 Visualization Accessibility - Who will think of the Users?

When evaluating the efficacy of a visual system, a core challenge, only truly tackled in recent years Kim et al. (2021), is the need for such visualizations to remain accessible to the visually impaired or even blind Elmqvist (2023) through, for example, sensory substitution Chundury et al. (2022). Here, instead of (solely) relying solely on visual channels to communicate data and information, non-visual channels, such as sound Zhao et al. (2008); Loeliger and Stockman (2014) or touch Taher et al. (2015); Holloway et al. (2018), can be used to make such systems more accessible and inclusive. Here, several members of the visualization community have made concerted efforts to communicate the importance of inclusivity as well as provide guidelines on how to ensure one's visualizations and visual system remain accessible Schimpf and Beddoes (2021); Firat and Laramee (2019); Osiobe et al. (2024). While such concerns may initially sound alien and strange to both metabolomics experts and developers alike, it is worth considering that, common especially among males, colorblindness is one of these visual impairments. Here, a small and easily implemented accessibility feature, both in visualizations for publication or interactive visualization systems, is usage of colorblind-friendly colormaps and scales Nelli (2024). Beyond the inclusive use of color, we strongly encourage especially developers to read these aforementioned reviews in order to think more generally about accessibility in their visualizations.

3 Dataset Descriptions

Brief Descriptions of the datasets used for generating figures.

1. Natural Product Discovery Dataset: olive solid mill waste mushroom study (Khatib et al. (2024)) was used. The study obtained LC-MS/MS data and has a multi-sample statistical design component.

2. Spectral data (ESI negative ionization mode) from an untargeted metabolomics study investigating the effects of nutrient starvation on maize plants was used (Othibeng et al., 2024, unpublised). Tools such as FBMN, IIMN and Spec2Vec were explored to show the effects of different scoring methods on network clustering. So far, we only zoomed-into clustering differences of HCAs across the different tools (but other clusters belonging to different classes can also be explored).

References

P. Chundury, B. Patnaik, Y. Reyazuddin, C. Tang, J. Lazar, and N. Elmqvist. Towards Understanding Sensory Substitution for Accessible Visualization: An Interview Study. *IEEE Transactions on Visualization and Computer Graphics*, 28(1):1084–1094, Jan. 2022. ISSN 1941-0506. doi: 10.1109/TVCG.2021.3114829. URL `https://ieeexplore.ieee.org/document/9552177`. Conference Name: IEEE Transactions on Visualization and Computer Graphics.

N. Elmqvist. Visualization for the Blind. *interactions*, 30(1):52–56, Jan. 2023. ISSN 1072-5520. doi: 10.1145/3571737. URL `https://doi.org/10.1145/3571737`.

E. E. Firat and R. S. Laramee. Inclusivity for visualization education: a brief History, investigation, and guidelines. *Diálogo com a Economia Criativa*, 4(12):146–160, Dec. 2019. ISSN 2525-2828. doi: 10.22398/2525-2828.412146-160. URL `https://dialogo.homologacao.emnuvens.com.br/revistadcec-rj/article/view/258`.

L. Holloway, K. Marriott, and M. Butler. Accessible Maps for the Blind: Comparing 3D Printed Models with Tactile Graphics. In *Proceedings of the 2018 CHI Conference on Human Factors in Computing Systems*, CHI '18, pages 1–13, New York, NY, USA, Apr. 2018. Association for Computing Machinery. ISBN 978-1-4503-5620-6. doi: 10.1145/3173574.3173772. URL `https://doi.org/10.1145/3173574.3173772`.

S. Khatib, I. Pereman, E. Kostanda, M. M. Zdouc, N. Ezov, R. Schweitzer, and J. J. J. van der Hooft. Olive mill solid waste induces beneficial mushroom-specialized metabolite diversity: a computational metabolomics study. *bioRxiv*, 2024. doi: 10.1101/2024.02.09.579616. URL `https://www.biorxiv.org/content/early/2024/02/09/2024.02.09.579616`.

N. W. Kim, S. C. Joyner, A. Riegelhuth, and Y. Kim. Accessible Visualization: Design Space, Opportunities, and Challenges. *Computer Graphics Forum*, 40(3):173–188, 2021. ISSN 1467-8659. doi: 10.1111/cgf.14298. URL `https://onlinelibrary.wiley.com/doi/abs/10.1111/cgf.14298`. _eprint: https://onlinelibrary.wiley.com/doi/pdf/10.1111/cgf.14298.

E. Loeliger and T. Stockman. Wayfinding without Visual Cues: Evaluation of an Interactive Audio Map System. *Interacting with Computers*, 26(5):403–416, Sept. 2014. ISSN 0953-5438. doi: 10.1093/iwc/iwt042. URL `https://doi.org/10.1093/iwc/iwt042`.

L. Nelli. Color Quest: An interactive tool for exploring color palettes and enhancing accessibility in data visualization. *PLOS ONE*, 19(3):e0290923, Mar. 2024. ISSN 1932-6203. doi: 10.1371/journal.pone.0290923. URL https://journals.plos.org/plosone/article?id=10.1371/journal.pone.0290923. Publisher: Public Library of Science.

E. U. Osiobe, S. Malallah, and N. E. Osiobe. Enhancing Data Visualization Accessibility: A Case for Equity and Inclusion, May 2024. URL https://papers.ssrn.com/abstract=4837601.

C. Schimpf and a. Beddoes. Designing equitable and inclusive visualizations: An underexplored facet of best practices for research and publishing. *Journal of Engineering Education*, 2021. URL https://onlinelibrary.wiley.com/doi/10.1002/jee.20388.

F. Taher, J. Hardy, A. Karnik, C. Weichel, Y. Jansen, K. Hornbæk, and J. Alexander. Exploring Interactions with Physically Dynamic Bar Charts. In *Proceedings of the 33rd Annual ACM Conference on Human Factors in Computing Systems*, CHI '15, pages 3237–3246, New York, NY, USA, Apr. 2015. Association for Computing Machinery. ISBN 978-1-4503-3145-6. doi: 10.1145/2702123.2702604. URL https://doi.org/10.1145/2702123.2702604.

H. Zhao, C. Plaisant, B. Shneiderman, and J. Lazar. Data Sonification for Users with Visual Impairment: A Case Study with Georeferenced Data. *ACM Trans. Comput.-Hum. Interact.*, 15(1):4:1–4:28, May 2008. ISSN 1073-0516. doi: 10.1145/1352782.1352786. URL https://doi.org/10.1145/1352782.1352786.
