# Supplementary figures and images for: Effective data visualization strategies in untargeted metabolomics
Source: Nat Prod Rep. 2024 Dec 2;42(6):982–1019. doi: 10.1039/d4np00039k (PMC11610048; doi:10.1039/d4np00039k)

Anscombe Dataset 1

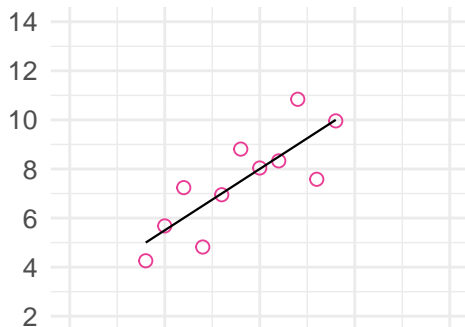

Anscombe Dataset 2

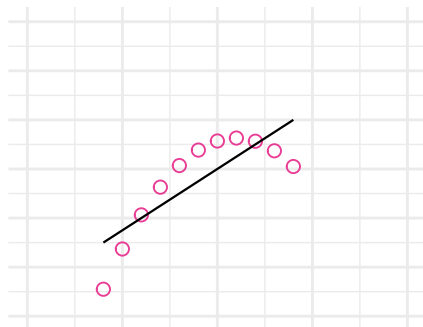

Anscombe Dataset 3

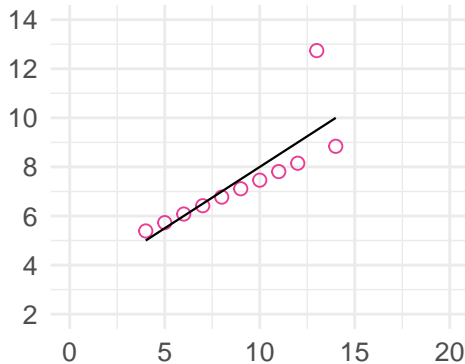

Anscombe Dataset 4

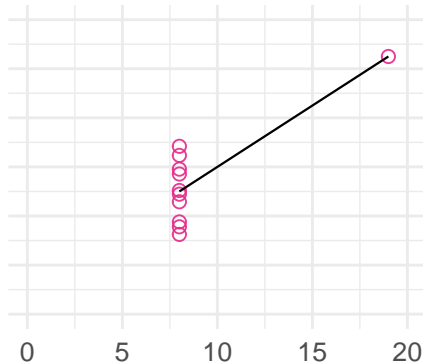

Supplement: NP-042-D4NP00039K-s002 [file NP-042-D4NP00039K-s002.zip › figures/anscombe.pdf]

# X-Shape

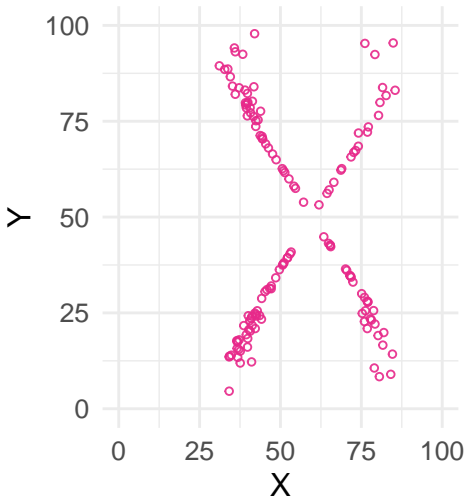

Supplement: NP-042-D4NP00039K-s002 [file NP-042-D4NP00039K-s002.zip › figures/dinosaur_dozen_x_shape.pdf]
